# Lipid metabolism and oxidative stress in patients with Alzheimer's disease and amnestic mild cognitive impairment

**DOI:** 10.1111/bpa.13202

**Published:** 2023-08-24

**Authors:** Yuting Nie, Changbiao Chu, Qi Qin, Huixin Shen, Lulu Wen, Yi Tang, Miao Qu

**Affiliations:** ^1^ Department of Neurology Xuanwu Hospital, Capital Medical University Beijing China

**Keywords:** Alzheimer's disease, biomarkers, lipid metabolism, oxidative stress

## Abstract

Lipid metabolism and oxidative stress are key mechanisms in Alzheimer's disease (AD). The link between plasma lipid metabolites and oxidative stress in AD patients is poorly understood. This study was to identify markers that distinguish AD and amnestic mild cognitive impairment (aMCI) from NC, and to reveal potential links between lipid metabolites and oxidative stress. We performed non‐targeted lipid metabolism analysis of plasma from patients with AD, aMCI, and NC using LC–MS/MS. The plasma malondialdehyde (MDA), glutathione peroxidase (GSH‐Px), and superoxide dismutase (SOD) levels were assessed. We found significant differences in lipid metabolism between patients with AD and aMCI compared to those in NC. AD severity is associated with lipid metabolites, especially TG (18:0_16:0_18:0) + NH4, TG (18:0_16:0_16:0) + NH4, LPC(16:1e)‐CH3, and PE (20:0_20:4)‐H. SPH (d16:0) + H, SPH (d18:1) + H, and SPH (d18:0) + H were high‐performance markers to distinguish AD and aMCI from NC. The AUC of three SPHs combined to predict AD was 0.990, with specificity and sensitivity as 0.949 and 1, respectively; the AUC of three SPHs combined to predict aMCI was 0.934, with specificity and sensitivity as 0.900, 0.981, respectively. Plasma MDA concentrations were higher in the AD group than in the NC group (*p* = 0.003), whereas plasma SOD levels were lower in the AD (*p* < 0.001) and aMCI (*p =* 0.045) groups than in NC, and GSH‐Px activity were higher in the AD group than in the aMCI group (*p* = 0.007). In addition, lipid metabolites and oxidative stress are widely associated. In conclusion, this study distinguished serum lipid metabolism in AD, aMCI, and NC subjects, highlighting that the three SPHs can distinguish AD and aMCI from NC. Additionally, AD patients showed elevated oxidative stress, and there are complex interactions between lipid metabolites and oxidative stress.

## Graphical abstract

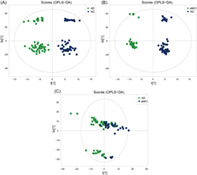

## 1 INTRODUCTION

The prevalence of dementia in China ranges from 4% to 7% and increases with age [1]. The annual per capita cost for patients with dementia in China is approximately 120,000 yuan, and the global estimates of costs for dementia is expected to exceed 2 trillion US dollars by 2030 [2]. The prevalence of dementia and its associated medical expenses will continue to increase as the population ages. It is well known that amyloid β (Aβ) deposition, vascular dysfunction, neurofibrillary tangles, synaptic dysfunction, and oxidative stress are the core pathogenic mechanisms of Alzheimer's disease (AD) [3]. The pathogenesis of AD is not fully explained by these pathways, and drugs targeting them have not had significant success. Currently, there is no specific drug that can cure dementia. Therefore, additional research on the pathogenesis and biomarkers of AD is required.

Lipidomics has become a hot topic in AD biomarkers, as lipids are the major macromolecular components of the brain and essential structural components of neuronal cell membranes. Lipids contribute to the processing of amyloid precursor proteins but also influence synaptogenesis, myelin formation, inflammation, oxidative stress, and other factors in the progression of AD pathology [4, 5]. A number of studies of lipid metabolism have been completed to identify potential markers for the diagnosis of AD, including long‐chain cholesteryl esters, ceramides (Cers), and sphingomyelin (SM) [6, 7]. However, some markers such as phosphatidylcholine (PC) and lysophosphatidylcholine (LPC) are inconsistent. For instance, in population of non‐Hispanic white, one study of AD patients found increased PC and LPC [8], while another cross‐sectional study found decreased levels of the five PCs and increased LPC in AD plasma samples [9]. In addition, a Netherlands study found unaltered PC concentrations and decreased LPC in AD [10]. Moreover, lipid metabolites that have been identified are difficult to replicate in various populations. Mapstone et al. identified 10 plasma lipid metabolites that can predict AD [11]. However, the results of this study were not replicated in the subsequent follow‐up of the Baltimore Longitudinal Study of Aging (BLSA) [12]. In most lipid metabolism studies, AD is primarily based on clinical symptoms, cognitive assessment and MRI diagnosis rather than amyloid imaging and cerebrospinal fluid (CSF) markers [7, 13, 14]. In the Chinese population, few studies have examined plasma lipid metabolism in AD or amnestic mild cognitive impairment (aMCI) using standard CSF biomarkers and amyloid imaging to diagnose AD [15]. Furthermore, few studies have simultaneously focused on lipid metabolism in AD, aMCI, and normal control (NC) groups.

Oxidative stress is a recognised and harmful factor in Alzheimer's disease [16]. The effects of oxidative stress on AD development include Aβ deposition, tau hyperphosphorylation, and neuronal loss [17]. Accumulated Aβ increases oxidative stress by disrupting the endogenous antioxidant system and increasing lipid peroxidation and protein oxidation, creating a vicious cycle [18, 19, 20]. Oxidative stress damage is widely present in AD [21, 22]. In AD patients, malondialdehyde (MDA) is widely present in brain tissue [23], and elevated MDA levels are also observed in the periphery serum of AD patients [24]. In addition, serum superoxide dismutase (SOD) was reduced in patients with mild cognitive impairment (MCI) and AD [21]. However, results regarding these oxidative stress markers in AD remain inconsistent [25].

A close link exists between lipid metabolites and oxidative stress. Understanding the association between lipid metabolites and oxidative stress can help to elucidate the mechanisms of metabolic disorders that lead to AD. In vitro, Cer has been reported to stimulate oxidative stress and induce neuronal death [26]. In animal models, cholesterol accumulation increases SOD activity and reduces neurodegenerative diseases [27]. In addition, feeding PC to mice increases SOD activity and decreases lipid peroxidation levels in the brain, which attenuates apoptosis and improves cognitive function [28]. However, the link between lipid metabolites and oxidative stress in plasma samples from patients is still poorly understood [29], as most studies are animal or in vitro studies. Therefore, the plasma lipid metabolism and oxidative stress of patients with AD must be examined comprehensively.

This study aimed to identify markers of AD and aMCI through untargeted lipid metabolomics. In addition, to further understand the link between oxidative stress and plasma lipid metabolism in AD, we assessed oxidative stress and lipid metabolism markers.

## 2 MATERIALS AND METHODS

### 2.1 Subjects

This study was conducted at Xuanwu Hospital of Capital Medical University and at the Guang Nei Community Health Service Center from January 2021 to June 2022. This study was approved by the Ethics Committee of Xuanwu Hospital, Capital Medical University (no. [2020] 097), which complies with the Declaration of Helsinki. This study was registered at ClinicalTrials.gov (registration number: ChiCTR2100041801). All the participants signed an informed consent form after being informed of the detailed study plan. AD and aMCI meet the National Institute on Aging‐Alzheimer's Association (NIA‐AA) working group's criteria [30, 31], and according to the evaluation of two neurologists. The threshold values of cerebrospinal fluid P‐tau/Aβ42 and the assay of cerebrospinal fluid were consistent with those previously reported [32]. Fifty AD patients had evidence of Aβ amyloid deposition, with decreased Aβ1‐42 in CSF or positive amyloid positron emission tomography (PET), and 9 AD patients met the core clinical diagnosis. Twenty aMCI patients had evidence of Aβ amyloid deposition, and 10 aMCI patients met the core clinical diagnosis. In addition, patients with AD had a clinical dementia score (CDR) ≥1, patients with aMCI had a CDR score of 0.5, with a memory score of at least 0.5. Each participant underwent neuropsychological evaluation, laboratory tests, and head MRI. The NC classification criteria included a Montreal Cognitive Assessment (MoCA) score ≥ 26 or Mini‐Mental State Examination (MMSE) score from 24 to 30, a CDR of 0, and no complaints of memory decline. This study excluded the following subjects: (1) those with a history of major depression or other mental disorders (schizophrenia, bipolar disorder, mania, etc.) within the past 2 years, or alcohol or drug abuse; (2) those with cognitive impairment from reversible causes, such as folic acid deficiency, vitamin B12 deficiency, hypothyroidism, or lupus encephalopathy; (3) patients with intracranial organic lesions, infections, or histories of cranial trauma or surgery; (4) those who underwent radiotherapy for malignancy within 3 years or with severe infections; (5) patients with long‐term steroid hormone use.

### 2.2 Neuropsychological assessment

We assessed the general cognitive function of the participants using the MMSE, the MoCA, and the CDR. The World Health Organization University of California‐Los Angeles Auditory Verbal Learning Test (WHO‐UCLA AVLT) was used to assess memory [33]. Attention was assessed using the Digit Span Forward test. The Trail‐Making Test and the Digit Span Backward Test were administered as measures of executive function [34]. The 30‐item Boston Naming Test (BNT) assessed language function and the Geriatric Depression Scale assessed emotional status. The severity of dementia is graded by the CDR score.

### 2.3 Lipidomics sample collection and methods

Metabolomic analysis was performed using a Nexera LC‐30A UHPLC system (Shimadzu, Japan) coupled with Q‐Exactive Plus (Thermo Scientific). Methanol and acetonitrile were purchased from Thermo Fisher Scientific. Formic acid and ammonium formate were purchased from Fluka and Sigma‐Aldrich, respectively. Plasma sample collection, sample preparation, quality control (QC) preparation, and LC–MS/MS mass spectrometry analysis procedures are shown in Supplementary Data 1.

### 2.4 Detection of oxidative stress

Glutathione peroxidase (GSH‐Px, E‐BC‐K096‐M) assay kits were purchased from Elabscience Biotechnology (Wuhan, China). Coefficient of variation (CV) for GSH‐Px was listed as below: intra‐assay CV: 2.4%; inter‐assay CV: 8.7%. Superoxide dismutase (SOD, A001‐3, intra‐assay CV: 5.05%; inter‐assay CV: 3.32%) and malondialdehyde (MDA, A003‐1, CV: 1.5%) assay kits were purchased from the Nanjing Jiancheng Bioengineering Institute (Nanjing, China). SOD and MDA levels were measured using WST‐1 and thiobarbituric acid methods, respectively, in strict accordance with the manufacturer's instructions.

### 2.5 Data analysis

Normally distributed continuous variables were expressed as mean ± standard deviation and categorical data as frequencies. Non‐normally distributed continuous data such as delayed recall, long‐delayed recognition, and MDA are shown as median and interquartile range (IQR). For normally distributed continuous variables, analysis of variance (ANOVA) was used to compare the participants' baseline characteristics between groups, followed by Tukey's honestly significant difference (HSD) post hoc multiple comparisons test, whereas chi‐square tests were used for categorical variables. The Kruskal–Wallis test was used for comparing non‐normally distributed data between the three groups, and the Steel–Dwass test was used for post hoc analysis. Comparison of three SPHs between AD and NC, aMCI and NC was performed using the Mann–Whitney *U* test. Correlations between oxidative stress marker concentrations, lipid metabolites and neuropsychological scores were assessed using partial correlation analysis (adjusted for education level, age, gender). Statistical significance was set at *p <* 0.05. Data analysis was performed using SPSS (IBM, Armonk, NY, USA; version 25.0). Potential biomarkers were calculated using receiver operating characteristic (ROC) curves. Calculation of the area under the curve (AUC), specificity and sensitivity of single and joint predictors using the ROCit package based on R4.2.3 software; the inverse probability weighting method was used to calculate single indicator‐corrected (adjusted for age, gender and education) AUC, specificity and sensitivity. Lipid metabolomics data processing demonstrated in Supplementary Data 2. The calculation of the sample size is shown in Supplementary Data 3. Hotelling's T2 test were used to examine outliers (Supplementary Figure S1).

## 3 RESULTS

### 3.1 Demographics

The basic demographics of all subjects are shown in Table 1. In the NC, aMCI, and AD, there were no significant differences in age, gender, education level, BMI, or prevalence of hypertension, diabetes, hyperlipidemia, stroke, or coronary heart disease. As expected, immediate recall, delayed recall, BNT, MMSE, MoCA, and long‐delayed recognition scores were lower in the aMCI and AD groups than in the NC group. We assessed the plasma levels of low‐density lipoprotein (LDL), high‐density lipoprotein (HDL), total cholesterol (TC), and triglyceride(TG), and all analyses showed no differences among the three groups.

**TABLE 1:** Demographic and clinical characteristics of study participants.

Variables	AD [59]	aMCI [30]	NC [54]	*p*‐Value
Age, years	64.07 ± 9.99	68.07 ± 8.78	66.20 ± 6.88	0.109
Education, years	10.85 ± 3.72	12.43 ± 3.43	12.07 ± 2.82	0.055
BMI (kg/m^2^)	23.51 ± 3.13	23.50 ± 2.75	24.72 ± 2.96	0.065
Gender(female), *n* (%)	36 (61.02%)	19 (63.33%)	34 (62.96%)	0.968
Hypertension, *n* (%)	19 (32.20%)	15 (50.00%)	23 (42.59%)	0.235
Diabetes, *n* (%)	6 (10.17%)	2 (6.67%)	7 (12.96%)	0.662
Hyperlipemia, *n* (%)	12 (20.34%)	6 (20.00%)	18 (33.33%)	0.216
Stroke, *n* (%)	4 (6.78%)	0 (0.00%)	4 (7.41%)	0.365
CHD, *n* (%)	4 (6.78%)	6 (20.00%)	3 (5.56%)	0.063
Aspirin intake, *n* (%)	7 (11.86%)	4 (13.33%)	10 (18.52%)	0.591
LDL (mmol/L)	2.77 ± 0.83	2.96 ± 0.71	2.90 ± 0.97	0.56
HDL (mmol/L)	1.37 ± 0.31	1.38 ± 0.26	1.43 ± 0.33	0.592
TC (mmol/L)	4.68 ± 1.00	4.91 ± 0.75	4.97 ± 0.92	0.212
TG (mmol/L)	1.46 ± 0.82	1.75 ± 0.75	1.73 ± 0.96	0.158
MMSE	17.69 ± 5.83^&^	25.70 ± 3.22^#^	28.37 ± 1.36*	<0.001
MoCA	12.92 ± 5.90^&^	21.33 ± 3.87^#^	25.72 ± 2.05*	<0.001
Immediate recall	13.24 ± 4.44^&^	19.17 ± 5.50^#^	27.33 ± 3.03*	<0.001
BNT	18.51 ± 4.75^&^	20.90 ± 4.23^#^	26.02 ± 2.27*	<0.001
Forward digit span	7.68 ± 1.27^&^	8.43 ± 1.14	8.19 ± 0.73*	0.003
Backward digit span	4.07 ± 1.38	4.70 ± 1.18	4.87 ± 0.99*	0.002
Delayed recall	0.00 (0.00–1.00)^&^	1.00 (1.00–5.50)^#^	10.00 (10.00–11.00)*	<0.001
Long‐delayed recognition	4.00 (2.50–6.00)^&^	6.00 (4.00–9.00)^#^	13.00 (13.00–13.00)*	<0.001

*Note*: One‐way ANOVA was used for comparing continuous variables among three groups, followed by Tukey's HSD post hoc multiple comparison test. The chi‐square test was used for comparing categorical variables among the three groups. The Kruskal–Wallis test was used for comparing non‐normally distributed data among the three groups, and the Steel–Dwass test was used for post hoc analysis.

Abbreviations: AD, Alzheimer's disease; aMCI, amnestic mild cognitive impairment; BMI: body mass index; CHD, coronary heart disease; HDL, high‐density lipoprotein; LDL, low‐density lipoprotein; MMSE, Mini‐Mental State Examination; MOCA, Montreal Cognitive Assessment; NC, normal control; TC, total cholesterol; TG, triglyceride.

*p < 0.05, AD versus NC; ^#^ *p* < 0.05, aMCI versus NC; ^&^ *p* < 0.05, AD versus aMCI.

### 3.2 Multivariate analysis of lipid metabolites

The results of the QC sample experiments showed good reproducibility, as the peak response intensities and retention times overlapped greatly (Supplementary Figure S2). All QC samples had correlation coefficients >0.9, indicating good reproducibility (Supplementary Figure S3). Multivariate control chart (MCC) showed good stability of the instrument (Supplementary Figure S4). Positive and negative ion modes detected 1519 lipid metabolites.

**FIGURE 1:**
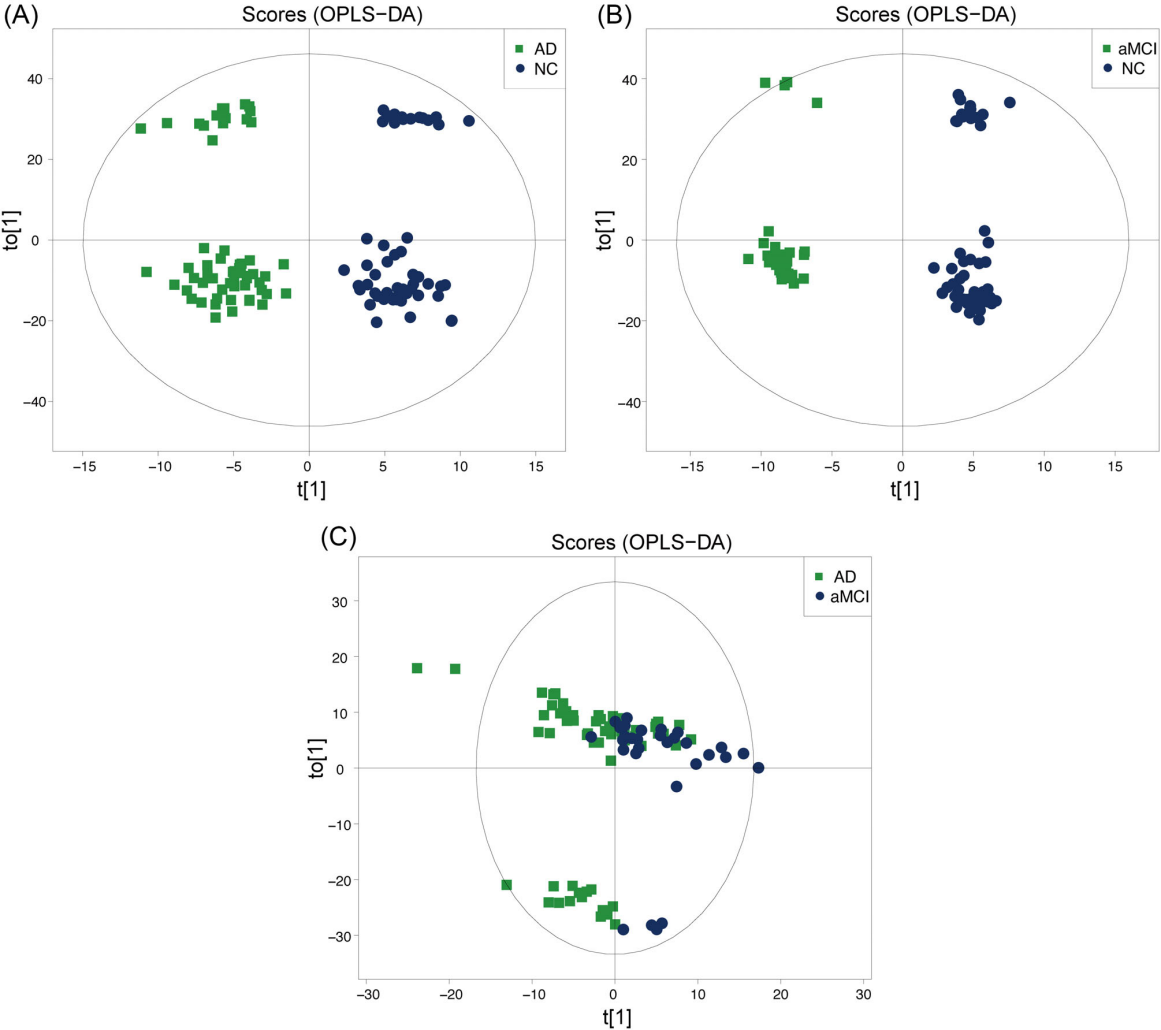
The score plot of the supervised orthogonal partial least squares discriminant analysis (OPLS‐DA). (A) OPLS‐DA score plot of AD versus NC. (B) OPLS‐DA score plot of aMCI versus NC. (C) OPLS‐DA score plot of AD versus aMCI.

**FIGURE 2:**
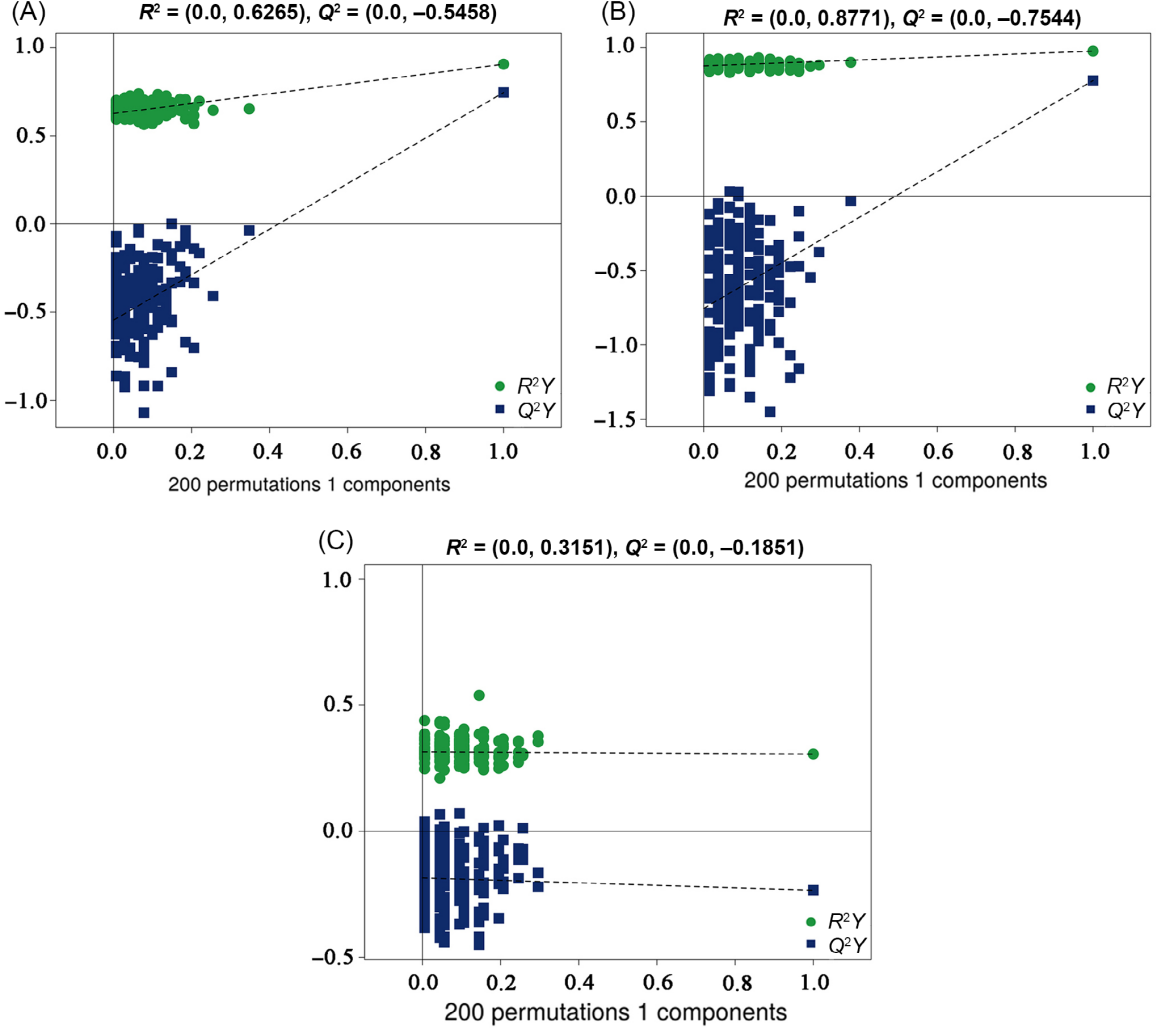
(A) Permutation test of the OPLS‐DA model for AD versus NC. (B) Permutation test of the OPLS‐DA model for aMCI versus NC. (C) Permutation test of the OPLS‐DA model for AD versus aMCI.

The OPLS‐DA showed significant changes in peripheral lipid metabolism in Chinese patients with AD or aMCI compared to the NC group (Figure 1A,B). There was a trend of separation between the AD and aMCI groups; however, there was partial overlap (Figure 1C). Supplementary Table S1 lists the parameters of the model evaluation (*R* ^2^ *Y*, *Q* ^2^). The validity of the model was evaluated using a permutation test. Figure 2A,B shows the permutation test of the OPLS‐DA model for AD and aMCI versus the NC group. The *R* ^2^ and *Q* ^2^ values tended to decrease as the replacement retention decreased, indicating that the model is robust and not over‐fitted. Figure 2C shows the overfitting phenomenon of the AD versus aMCI OPLS‐DA model replacement test plot.

### 3.3 Different plasma lipid metabolites profiles in the AD and NC groups

**FIGURE 3:**
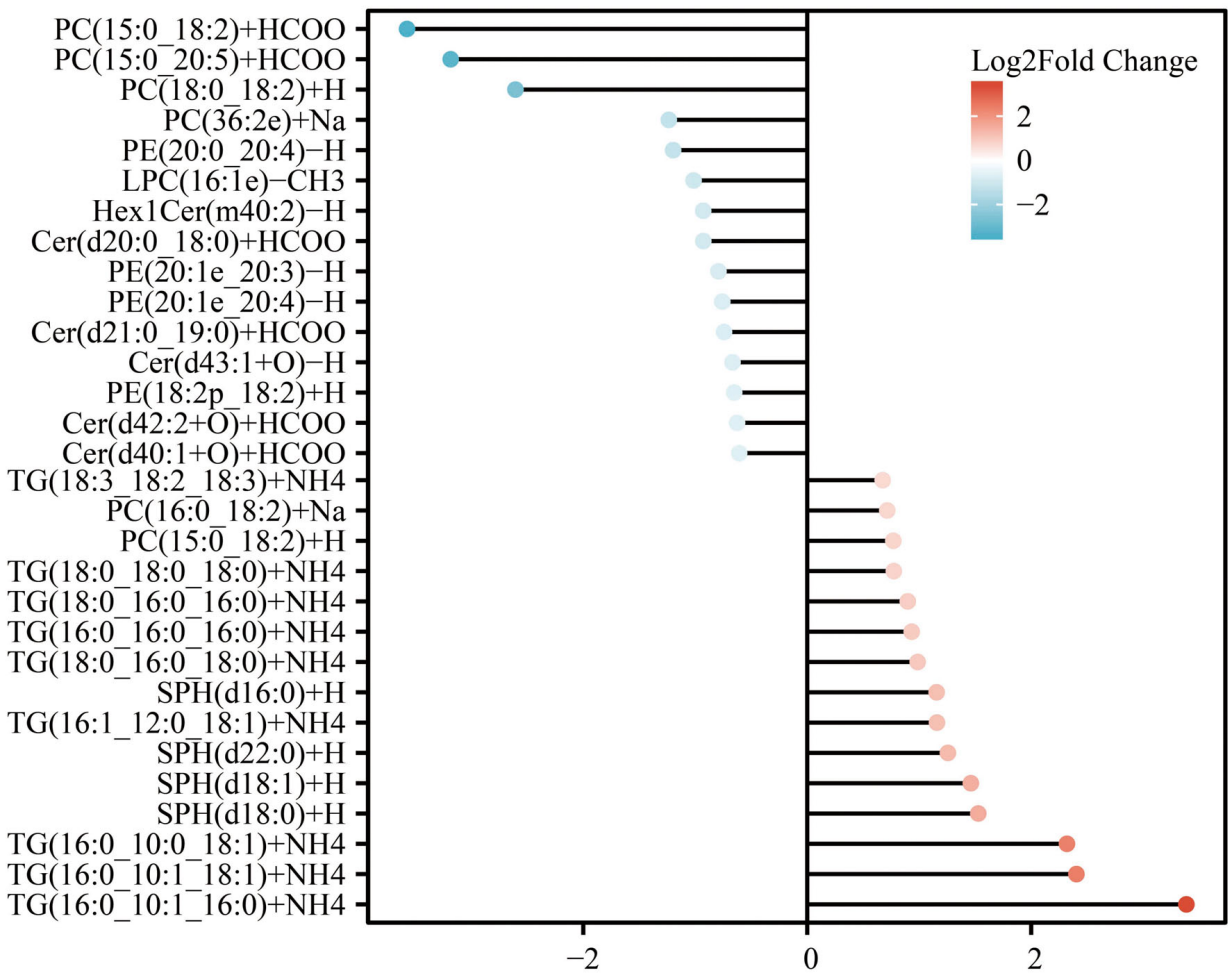
Different lipid metabolites of AD and NC. Values of dots indicate the log2 fold change values, red indicates up‐regulation of lipid metabolites and blue indicates down‐regulation of lipid metabolites. Cer, ceramides; Hex1Cer, hexosyl ceramide; LPC, lysophosphatidylcholine; LPE, lysophosphatidylethanolamine; PC, phosphatidylcholines; PE, phosphatidylethanolamines; SM, sphingomyelin; SPH, sphingosine; TG, triglyceride.

In total, 79 lipid metabolites were identified based on VIP >1 and independent samples *t*‐test (*p* < 0.05) (Supplementary Table S2). After controlling for covariates, lipid metabolites that were significantly differentially expressed (VIP >1, *p <* 0.05, FC >1.5, or <0.67) were selected for further analysis (Figure 3, Supplementary Figure S5A). Two PCs, nine TGs, and four sphingosines (SPHs) were significantly up‐regulated in AD patients, whereas five Cers, four phosphatidylethanolamines (PEs), four PCs, one hexosyl ceramide (Hex1Cer), and one LPC were significantly down‐regulated in AD subjects. In addition, the results after covariate control were shown in Supplementary Table S3.

### 3.4 Different plasma lipid metabolites profiles in the aMCI and NC groups

**FIGURE 4:**
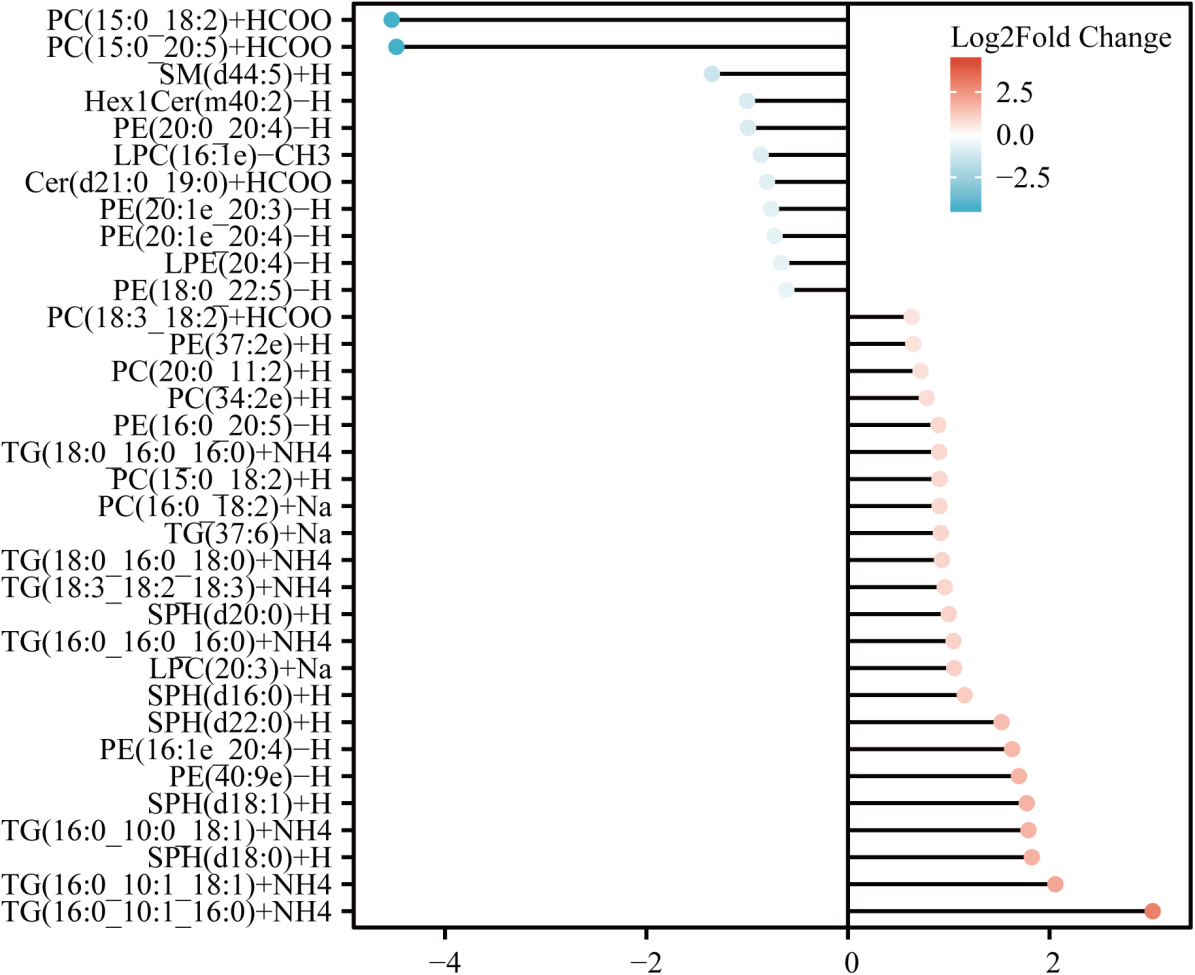
Different lipid metabolites of aMCI and NC. The value of the dot indicates the log2 fold change values, with red indicating that lipid metabolites are up‐regulated and blue indicating that lipid metabolites are down‐regulated.

Seventy‐six lipid metabolites that distinguished patients with aMCI from healthy individuals were identified using the OPLS‐DA model (Supplementary Table S4). After controlling for covariates, lipid metabolites that were significantly differentially expressed (VIP >1, *p* < 0.05, FC >1.5, or <0.67) were selected for further analysis (Figure 4, Supplementary Figure S5B). Five PCs, eight TGs, four PEs, five SPHs, and one LPC were significantly up‐regulated in patients with aMCI, whereas two PCs, one Cer, one SM, one Hex1Cer, four PEs, one lysophosphatidylethanolamine (LPE), and one LPC were significantly down‐regulated. Interestingly, we found similar changes in lipid metabolism in the aMCI and AD groups, suggesting that the lipid metabolism of aMCI patients was altered during the pre‐AD period. The results after controlling for covariates are shown in Supplementary Table S5.

### 3.5 Altered lipid metabolites with AD severity

**FIGURE 5:**
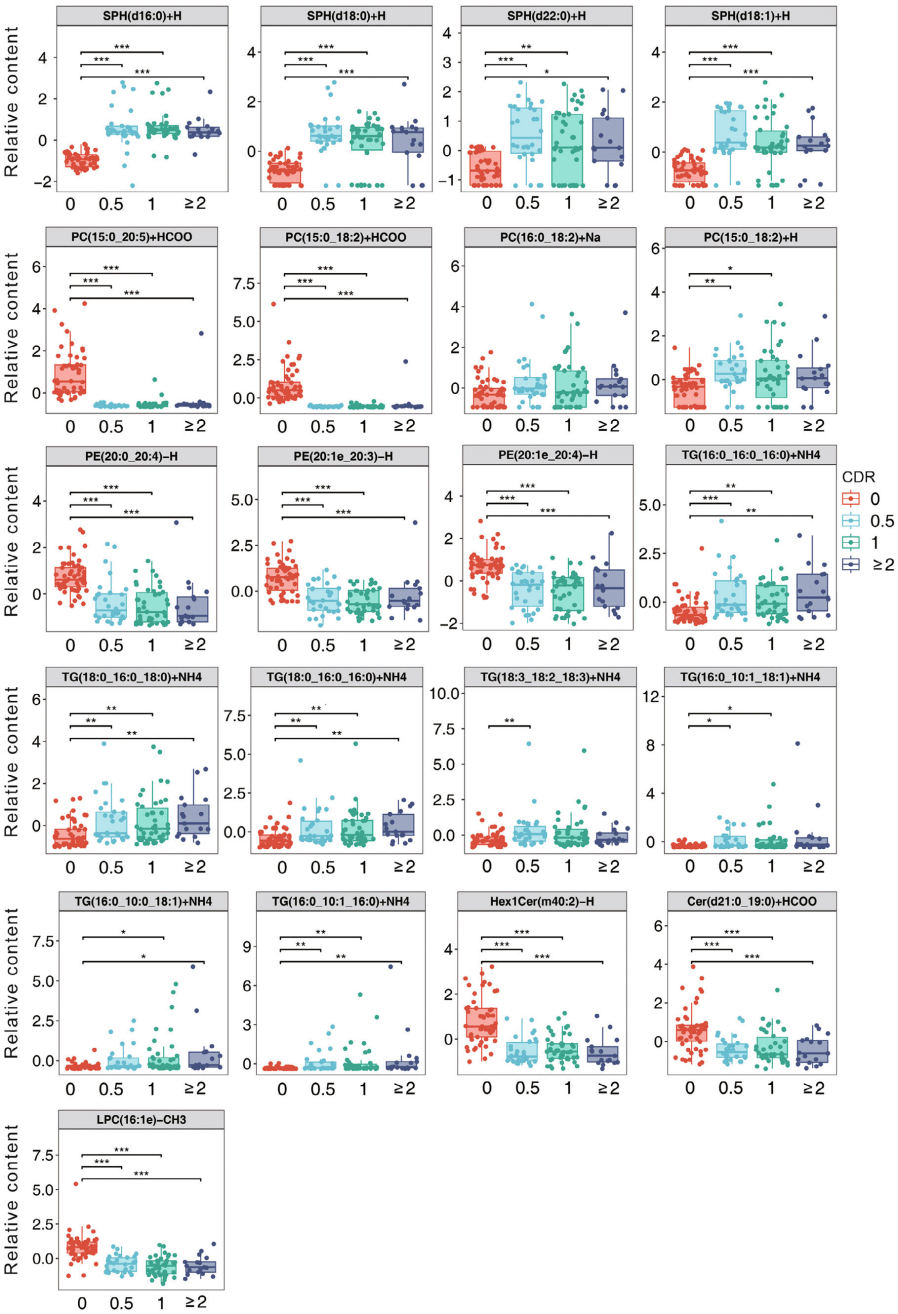
Box plot of 21 lipid metabolites based on CDR scores. The *p*‐value was calculated using Kruskal–Wallis test. *p < 0.05; **p < 0.01; ***p < 0.001.

We plotted box plots of significantly different shared lipid metabolites based on CDR scores (Figure 5). Comparison of lipid metabolites by CDR grading showed that progression from mild to moderate–severe AD was accompanied by changes in lipid metabolites. The relative content of TG (18:0_16:0_18:0) + NH4, TG (18:0_16:0_16:0) + NH4 showed an increasing trend with disease severity, while PE (20:0_20:4)‐H, LPC (16:1e)‐CH3 showed a tendency to decrease with the aggravation of the disease.

### 3.6 Identification of lipid markers for AD and aMCI

**FIGURE 6:**
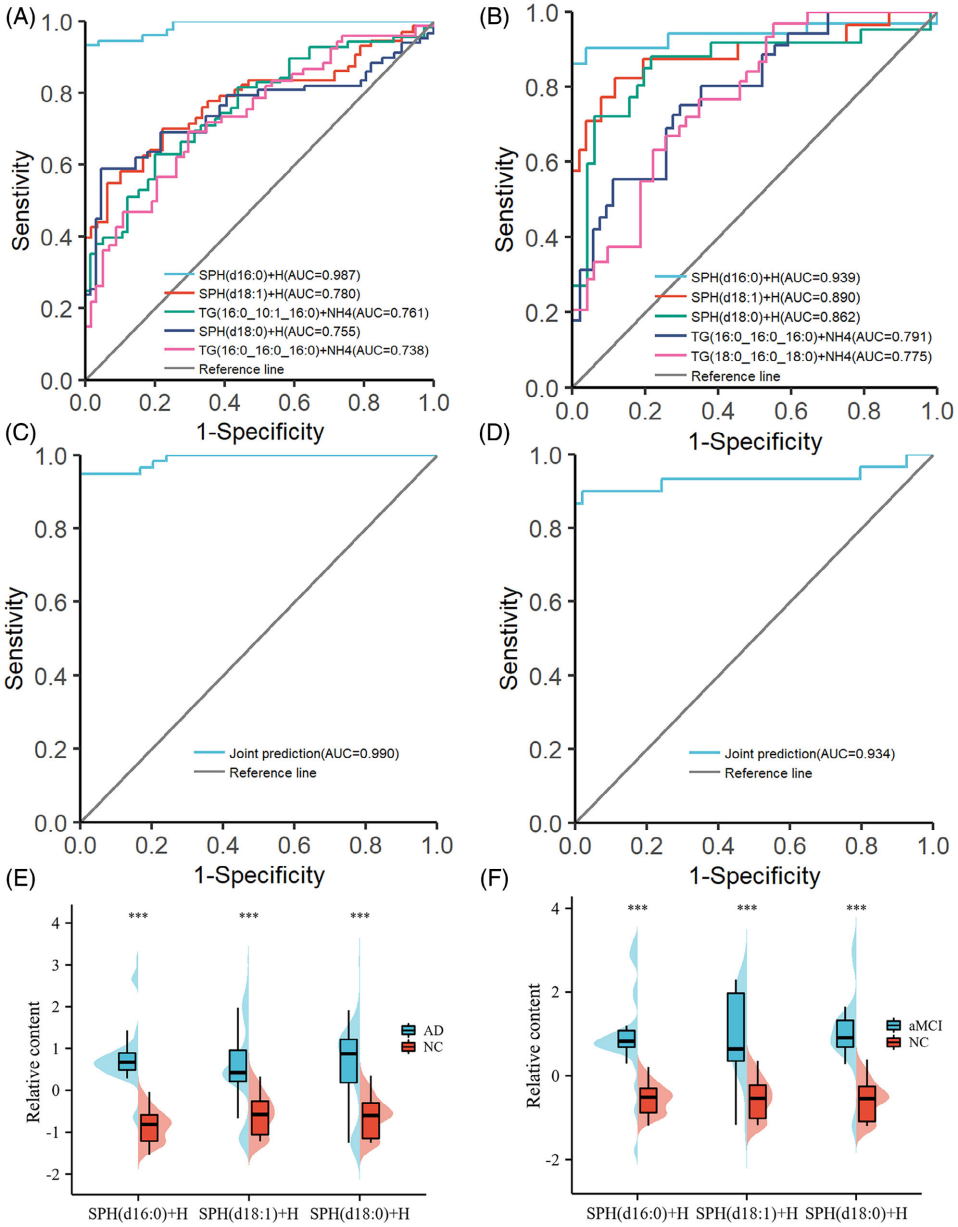
(A) Receiver operating characteristic (ROC) curve used to differentiate between AD and NC subjects. (B) ROC curve used to differentiate between aMCI and NC subjects. (C) ROC curves of the three SPHs (SPH (d16:0) + H, SPH (d18:1) + H, and SPH (d18:0) + H) for joint prediction of AD. (D) ROC curves of the three SPHs (SPH (d16:0) + H, SPH (d18:1) + H, and SPH (d18:0) + H) for joint prediction of aMCI. (E) Comparison of three SPHs between AD and NC. (F) Comparison of three SPHs between aMCI and NC. The Mann–Whitney *U* test was used to compare the two groups, *p < 0.05; **p < 0.01; ***p < 0.001.

To assess the diagnostic performance of plasma lipid metabolites for AD and aMCI, we performed ROC analysis for significant lipid metabolites with VIP >1.0, FC >1.5, *p* < 0.05 (Figure 6A–F, Supplementary Tables S6–S11). Among the lipid metabolites distinguishing the NC and AD groups, the AUC of SPH (d16:0) + H, SPH (d18:1) + H, and SPH (d18:0) + H, was 0.987 (0.961–1.000), 0.780 (0.680–0.853), and 0.755 (0.629–0.839), respectively; in addition, TG (16:0_10:1_16:0) + NH4 and TG (16:0_16:0_16:0) + NH4 also had good discriminatory potential. Among the lipid metabolites distinguishing the NC and aMCI groups, the AUC areas of SPH (d16:0) + H, SPH (d18:1) + H, and SPH (d18:0) + H were 0.939 (0.843–1.000), 0.890 (0.737–0.962), 0.862 (0.689–0.945), respectively; in addition, TG (16:0_16:0_16:0) + NH4 and TG (18:0_16:0_18:0) + NH4 also had good discriminatory potential. SPH (d16:0) + H, SPH (d18:0) + H, and SPH (d18:1) + H were able to distinctly distinguish AD and aMCI from NC. Next, we combined three SPHs to predict AD and aMCI, respectively, and found that the AUC of three SPHs combined to predict AD was 0.990 (0.974–1.000), with specificity and sensitivity of 0.949,1, respectively; the AUC of three SPHs combined to predict aMCI was 0.934 (0.860–0.999), with specificity and sensitivity 0.900, 0.981, respectively.

### 3.7 Oxidative stress markers in the AD and aMCI groups

**FIGURE 7:**
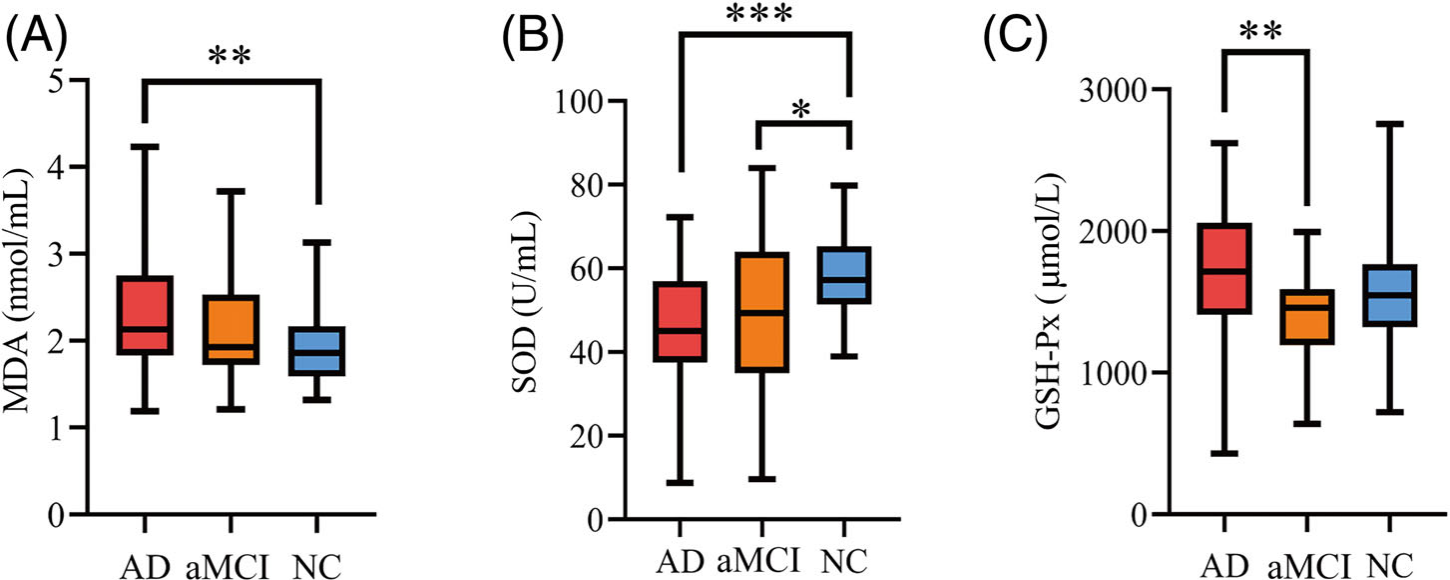
(A) MDA concentrations in plasma. (B) SOD activity in plasma. (C) GSH‐Px activity in plasma. GSH‐Px; glutathione peroxidase; MDA; malondialdehyde; SOD; superoxide dismutase.

We measured MDA, SOD, and GSH‐Px (Figure 7A–C, Table 2). We found that MDA concentrations were higher (*p =* 0.003) and SOD activity was lower (*p <* 0.001) in AD group than in NC. AMCI group had a lower SOD activity than NC group (*p =* 0.045). GSH‐Px was lowest in aMCI group compared to AD (*p =* 0.007), but no difference was observed compared to that in the NC group.

**TABLE 2:** Alterations in oxidative stress markers in AD and aMCI.

Variables	AD	aMCI	NC	*p*
MDA, nmol/mL	2.13 (1.83–2.70)*	1.92 (1.74–2.52)	1.86 (1.60–2.15)	0.005
SOD, U/mL	45.81 ± 13.02*	50.75 ± 18.80^#^	58.09 ± 9.63	<0.001
GSH‐Px, μmol/L	1685.25 ± 464.12^&^	1403.72 ± 338.82	1583.59 ± 380.43	0.011

Abbreviations: AD, Alzheimer's disease; aMCI, amnestic mild cognitive impairment; GSH‐Px, glutathione peroxidase; MDA, malondialdehyde; NC, normal control; SOD, superoxide dismutase.

*p < 0.05, AD versus NC; ^#^ *p <* 0.05, aMCI versus NC; ^&^ *p <* 0.05, AD versus aMCI.

### 3.8 Correlations between altered lipid metabolites and oxidative stress

**FIGURE 8:**
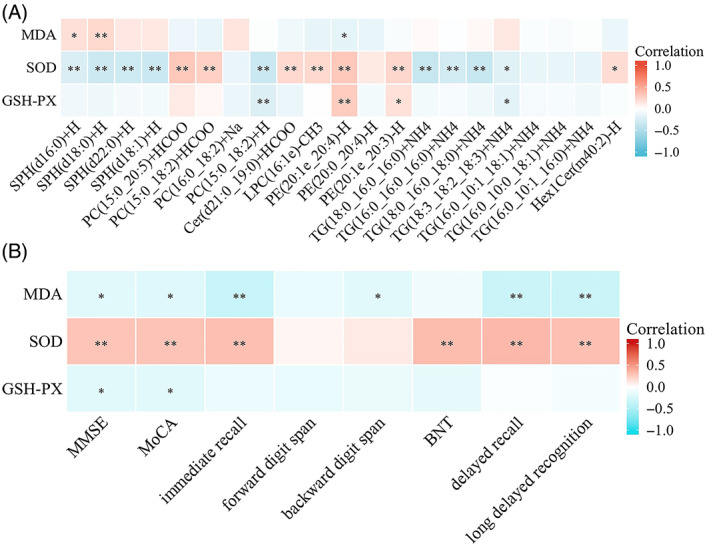
(A) Correlation of co‐altered lipid metabolites with oxidative stress. (B) Correlation of oxidative stress markers with cognitive function. Warm colours (red) are positively correlated and cool colours (blue) are negatively correlated. Adjusted for age, gender and education level. BNT, Boston Naming Test. *p < 0.05; **p < 0.01.

We also evaluated the correlation between the altered lipid metabolites and oxidative stress levels (Figure 8A, Supplementary Table S12). After adjusting for potential confounders, two SPHs positively correlated with the oxidative stress marker MDA, and one PE was negatively associated with MDA. Four SPHs, one PC, and four TGs were negatively correlated with SOD, and the lipid metabolites positively correlated with SOD, including two PEs, two PCs, one LPC, and one Hex1Cer. Lipid metabolites that were positively associated with GSH‐Px included two PEs, and one PC, one TG negatively associated with GSH‐Px. These results suggest that disturbances in plasma lipid metabolites are associated with alterations in oxidative stress or antioxidant enzyme systems.

### 3.9 Correlation between oxidative stress markers and cognitive function

We investigated the correlation between oxidative stress markers and cognitive scores (Figure 8B, Supplementary Table S13). After adjusting for potential confounders, SOD positively correlated with long‐delayed recognition (*r* = 0.308, *p <* 0.001), delayed recall (*r* = 0.338, *p <* 0.001), immediate recall (*r* = 0.308, *p <* 0.001), BNT scores (*r* = 0.324, *p <* 0.001), MoCA scores (*r* = 0.291, *p <* 0.001), and MMSE scores (*r* = 0.284, *p* < 0.001). The MDA concentration was negatively correlated with long‐delayed recognition (*r* = −0.332, *p* < 0.001), delayed recall (*r* = −0.332, *p <* 0.001), immediate recall (*r* = −0.348, *p* < 0.001), backward digit span scores (*r* = −0.192, *p =* 0.022), MoCA scores (*r* = −0.210, *p =* 0.012), and MMSE scores (*r* = −0.192, *p =* 0.022). GSH‐Px was negatively correlated with MoCA scores (*r* = −0.193, *p =* 0.021), and MMSE scores (*r* = −0.192, *p =* 0.021).

## 4 DISCUSSION

To our knowledge, few studies have examined the peripheral lipid metabolism in patients with biomarker‐confirmed AD or aMCI, and more than two‐thirds of the AD and aMCI patients in our study were confirmed by CSF or PET. In this study, the lipid metabolism profile of AD and aMCI patients was significantly different from that of NC, and the three SPHs were effective in differentiating AD and aMCI from NC. The process of cognitive decline in AD is accompanied by alterations in lipid metabolites, in particular TG (18:0_16:0_18:0) + NH4, TG (18:0_16:0_16:0) + NH4, PE (20:0_20:4)‐H and LPC (16:1e)‐CH3. In addition, in our study, oxidative stress was elevated and antioxidant capacity was reduced in the peripheral blood of the AD group. Correlation analysis revealed that lipid metabolites were significantly correlated with oxidative stress levels.

According to most studies [8, 35, 36], TG levels are higher in patients with AD or cognitive impairment. A recent non‐targeted lipidomics study included 94 AD cases and 64 controls and highlighted in the results that plasma TGs were significantly elevated in AD patients [8]. A cross‐sectional study examined the lipid profile of 273 subjects (183 cognitively impaired; 90 cognitively healthy) and found that serum TG levels were significantly higher in cognitively impaired patients [35]. A study in a Mexican population found that TG levels were higher in AD patients and that TG concentrations increased progressively with cognitive decline [36]. In general agreement with most studies, our data showed elevated serum TG levels in AD and aMCI group compared with NC. However, studies by others have reported reduced TG levels in AD [37]. The difference may be caused by the fact that the study used a common standard enzyme colorimetric technique to detect TG levels. In addition, elevated serum TG levels accelerated cognitive decline and were associated with increased brain Aβ deposition [38, 39, 40]. Overall, TG is considered to be a dangerous factor for AD and may accelerate cognitive impairment.

In our study, PC lipid metabolites in AD and aMCI group showed conflicting performance, with some PC metabolites increasing and others decreasing. Most previous lipid metabolism studies have reported decreased PC levels in AD or MCI [41, 42, 43, 44]. Lower PC levels are associated with decreased memory and reduced intracortical neuronal activity in normal ageing, particularly in the internal olfactory cortex [45]. However, other studies have reported elevated PC levels in AD. For example, a large cross‐sectional study found that two PCs lipid species were increased in AD [46]. Another longitudinal study over a 7–9 year period found a four PCs increase during the MCI phase and a five PCs increase when cognitively healthy individuals converted to AD [47]. The discrepancy may be caused by the use of inconsistent lipidomic assay techniques and platforms, in addition to the inclusion of more patients with mild AD in the two studies mentioned above (mean MMSE score of 20), in addition to the long follow‐up period resulting in changes in lipids in plasma samples. A small sample cross‐sectional study supported our results, reporting conflicting PC lipid molecule results and suggesting that such conflicting results may be related to fatty acid saturation [48]. Thus, the physiological functions of different PC lipid species may be completely opposite.

Our study found that blood PE levels decreased in patients with AD. The available literature provides conflicting evidence regarding the expression of PE in AD and MCI. Many autopsy studies have reported reduced PE levels in AD patients [49, 50]. A reduction in serum PE levels was also observed in AD patients [48, 51]. Reduced brain PE was observed in mice with significantly impaired cognitive function [52]. In contrast, some studies have shown increased PE concentrations in subjects with AD or MCI and established a strong correlation between PE and the degree of brain atrophy [53, 54]. However, both studies had small sample sizes (subjects <30) and did not use CSF or PET to confirm the diagnosis of AD and MCI. Decreased PE in AD may be caused by oxidative stress, as reported in previous in vitro studies, and oxidative stress could cause a dramatic decrease in PE concentration [55]. In our study, all the patients with AD and MCI exhibited high levels of oxidative stress.

Here we found that LPC was down‐regulated in AD patients. Our findings are supported by several studies of plasma and CSF samples, which showed reduced LPC concentrations in AD group compared to NC group [10, 56]. In contrast, an earlier study found that patients with AD and MCI had higher plasma levels of LPC [9]. However, this study only included patients older than 70 years with milder degrees of AD (mean MMSE score of 20). The inconsistent LPC levels in AD may be caused by the different confounding factors involved or the different AD severities. In conclusion, LPC in AD and aMCI requires further investigation.

Our findings show elevated plasma SPH levels in AD and aMCI group. Our study identified three SPHs that can differentiate AD from aMCI. Our results are supported by several studies on brain tissue samples. An autopsy study showed elevated levels of SPH in AD brains [57]. Similarly, He et al. found elevated SPH levels in brains with AD [58]. SPH induces apoptosis in hippocampal neurons [59, 60]. In addition, several studies have recently reported that SPH accumulation leads to demyelination by causing apoptosis of oligodendrocytes [61, 62, 63]. Notably, Oligodendrocytes are primarily responsible for myelin formation, demyelination and oligodendrocyte dysfunction are present in the brains of early AD mice [64, 65], and myelin loss precedes amyloid and tau pathology [66]. In addition, autopsy and neuroimaging evidence from the brains of AD patients suggests that there is extensive myelin damage in the brains of AD patients [67, 68, 69]. According to recent studies, AD mice treated with drugs that promote myelin renewal have increased hippocampal neuronal activity and improved spatial memory [70]. These studies suggest that inhibition of SPH production, reduction of oligodendrocyte apoptosis and promotion of myelin regeneration may be one of the treatments for AD.

Cer is involved in multiple pathways in AD pathology, including tau protein phosphorylation, apoptosis, oxidative stress, and Aβ deposition [71, 72]. Most previous studies have shown significant increases in Cer levels in AD patients [7, 73, 74]. However, in our study, multiple Cer concentrations were reduced in AD and aMCI group. The reason for the difference in results may be that the above‐mentioned studies included subjects of different ages and at different levels of severity of AD. Our results are supported by a longitudinal study, which showed that Cer levels were higher when memory was normal but lower after the onset of memory impairment, and that Cer levels varied with the duration and severity of cognitive impairment [75]. Our results are supported by another cross‐sectional study that found lower plasma Cer levels in the MCI group than in the NC group [76]. A possible explanation is that Cer concentration fluctuates as the disease progresses during AD development. Furthermore, in an earlier in vitro study, high levels of glutathione inhibited ceramide production [77]. GSH‐PX levels were high in AD group in our study, which could be one of the reasons for the decrease in Cer.

AD and aMCI patients showed changes in peripheral oxidative stress markers. In our study, MDA was grown up in the AD group compared to the NC group, while SOD concentrations decreased in both the AD and aMCI groups. Most studies have shown that circulating MDA concentrations are elevated in patients with AD, which is consistent with previous studies [78, 79]. However, additional studies have shown that serum MDA concentrations in AD group were unchanged compared to the NC [80, 81]. Some studies have reported reduced [21, 82], increased [83], or normal [84] SOD levels in the erythrocytes or serum of AD patients. In addition, we found elevated serum GSH‐Px activity in patients with AD, possibly because of a compensatory mechanism of the disease, which is consistent with previous findings [85, 86]. However, other studies have observed that patients with AD have lower GSH‐Px [87, 88] or normal GSH‐Px [24, 89]. The discrepancy in the results could be caused by the use of less stringent criteria or the lack of records regarding antioxidant‐related drug administration.

A positive correlation was found between SOD and cognitive function in this study, whereas MDA was negatively correlated with cognitive function. Most studies have confirmed that MMSE is negatively correlated with MDA levels in patients with AD [78, 88], which is consistent with our results. Furthermore, our results are supported by a study on human postmortem brain tissue samples that demonstrated a positive correlation between MMSE and SOD activity [90]. However, a cohort study in China reported a negative association between plasma SOD activity and cognitive function [91]. Nonetheless, the participants involved in the study were not AD patients but the general population of older adults in the community.

The relevance of SPH to oxidative stress is particularly important because these three SPHs are markers that distinguish AD and aMCI from the NC group. Our study demonstrated that the correlation between SPH and MDA was positive, and the correlation between SPH and SOD was negative. This finding was confirmed by an in vitro study, in which SPH significantly increased oxidative stress levels in rat retinal neurons [92]. Furthermore, TGs levels were negatively correlated with SOD levels in our study. This was previously demonstrated in a study of a healthy population in which a high‐fat diet led to elevated plasma TG levels and disrupted circulating antioxidant defences [93]. In a subsequent clinical study, a decrease in plasma SOD activity was observed with an increase in TGs [94]. PE was positively correlated with SOD and GSH‐Px, and negatively correlated with MDA. EPA‐enriched phosphatidylethanolamine (EPA‐PE) was used to feed AD mice, and EPA‐PE increased SOD activity, decreased MDA levels [95]. In this study, multiple PCs were significantly positively correlated with SOD and negatively correlated with MDA. In preclinical studies, PC increased SOD activity in the mouse brain, and reduced cognitive decline [96]. In conclusion, oxidative stress and lipid metabolism have been closely linked in vitro and in animals. However, except for TG, there is limited evidence of a correlation between human peripheral plasma PE, PC, and SPH and oxidative stress, and our study provides evidence for the relationship between peripheral lipids and oxidative stress.

This is the first study to correlate lipid metabolism with oxidative stress in the peripheral blood of Chinese AD or aMCI patients. Our study has many strengths. Plasma samples from more than two‐thirds of the AD/aMCI participants in our study were confirmed by CSF or PET biomarkers to ensure specificity and accuracy of diagnosis. We also recruited a larger sample of AD, aMCI and NC patients. There were no differences in baseline levels of LDL, HDL, TC, and TG among the three groups, indicating that lipid nutritional status was consistent across all study groups. However, our study has some limitations. First, APOE‐ε4 was an important covariate that was not considered in this study. Second, we focused only on cross‐sectional results, and therefore could not determine whether the metabolite changes were pathogenic or secondary to disease‐related processes. Longitudinal studies of dementia metabolites would clarify causal inferences. In addition, the differential metabolites identified in our study require animal experiments to verify causality and absolute quantitative assays using the target metabolic histology. Finally, future studies could use cerebrospinal fluid samples to validate the discriminatory potential of SPH for AD.

## 5 CONCLUSION

Overall, our results suggest that lipid metabolism is abnormal in AD and aMCI group, and that three SPHs as high‐performance biomarkers can clearly distinguish AD and aMCI from NC. In addition, our study confirmed increased oxidative stress in patients with AD and aMCI and revealed a potential link between lipid metabolites and oxidative stress. Additionally, a correlation was observed between enhanced oxidative stress (especially MDA) and cognitive decline.

## AUTHOR CONTRIBUTIONS

Miao Qu and Yi Tang were responsible for study design. Yuting Nie and Changbiao Chu analysed the data and wrote the manuscript. Yuting Nie, Changbiao Chu, Qi Qin, Huixin Shen and Lulu Wen were responsible for recruiting the subjects and collecting clinical data. Miao Qu, Qi Qin and Yi Tang reviewed the manuscript. All the authors have read and agreed to the published version of the manuscript.

## CONFLICT OF INTEREST STATEMENT

The authors declare no conflicts of interest.

## INSTITUTIONAL REVIEW BOARD STATEMENT

Informed consent was obtained from all subjects involved in the study.

## INFORMED CONSENT STATEMENT

This study was approved by the Ethics Committee of Xuanwu Hospital, Capital Medical University (no. [2020] 097), which complies with the Declaration of Helsinki.

## Supporting information


**Supplementary Data 1.** Lipidomics sample collection and LC‐MS/MS analysis.
**Supplementary Data 2.** Lipid metabolomics data processing.
**Supplementary Data 3.** Lipid metabolomics sample size calculation.

**Supplementary Figure S1.** The horizontal coordinates represent all experimental and QC samples, the vertical coordinates reflect confidence intervals, and the red line defines the 99% confidence interval range.
**Supplementary Figure S2.** The horizontal coordinates of the graph indicate the retention time of each peak and the vertical coordinates indicate the intensity values of the peaks. QC sample experimental results showed that the chromatographic peak response intensity and retention time of QC samples basically overlapped, indicating that the experimental repeatability was good. (A) Base Peak chromatogram (BPC) overlapping of all QC samples in negative ion mode. (B) Base Peak chromatogram (BPC) overlapping of all QC samples in positive ion mode.
Pearson correlation analysis was performed on the QC samples. A general correlation coefficient greater than 0.9 indicates a good correlation. The experimental results showed that the correlation coefficients between QC samples were all above 0.9, indicating that the experimental repeatability was excellent.
**Supplementary Figure S3.** The abscissa and ordinate in the figure represent each QC sample. The points in each cell represent the ion peaks (metabolites) extracted from the QC samples, and the abscissa and ordinate represent the log values of the ion peak signal intensity values.
Multivariate Control Chart (MCC) is a multivariate statistical model established based on the ion peaks detected in QC samples, and is a quality management tool used to monitor and judge whether the instrument status is stable. Each point in the multivariate control chart represents a QC sample, and the X‐axis is the order in which all QC samples were loaded. The points in the graph fluctuate up and down because of fluctuations in the state of the instrument. Generally, the normal range is within plus or minus 3 standard deviations. The experimental results showed that the fluctuation of QC samples was within the range of plus or minus 3 standard deviations, reflecting that the fluctuation of the instrument was within the normal range, and the data could be used for subsequent analysis.
**Supplementary Figure S4.** The abscissa represents each QC sample, the ordinate reflects the standard deviation, and the yellow and red lines define a range of plus or minus 2 and 3 standard deviations, respectively.
**Supplementary Figure S5.** (A) The variable important in projection (VIP) score of AD and NC. (B) VIP score of aMCI and NC.

**Supplementary Table S1.** Evaluation parameters of OPLS‐DA model.
**Supplementary Table S2.** Lipid metabolites altered in plasma of AD versus NC.
**Supplementary Table S3.** Lipid metabolites altered in plasma of AD versus NC (analysis of covariance).
**Supplementary Table S4.** Lipid metabolites altered in plasma of aMCI versus NC.
**Supplementary Table S5.** Lipid metabolites altered in plasma of aMCI versus NC (analysis of covariance).
**Supplementary Table S6.** Identification of potential markers for AD (adjusted for none).
**Supplementary Table S7.** Identification of potential markers for AD (adjusted for age, gender, education level).
**Supplementary Table S8.** ROC analysis of SPH(d16:0) + H, SPH(d18:1) + H, and SPH(d18:0) + H in the diagnosis of AD.
**Supplementary Table S9.** Identification of potential markers for aMCI (adjusted for none).
**Supplementary Table S10.** Identification of potential markers for aMCI (adjusted for age, gender, education level).
**Supplementary Table S11.** ROC analysis of SPH(d16:0) + H, SPH(d18:1) + H, and SPH(d18:0) + H in the diagnosis of aMCI.
**Supplementary Table S12.** Correlation between lipid metabolites and oxidative stress.
**Supplementary Table S13.** Correlation between lipid metabolites and oxidative stress.

## Data Availability

Data from this study are provided in the supplementary document.
