# Supplementary material for: Lipid metabolism and oxidative stress in patients with Alzheimer's disease and amnestic mild cognitive impairment
Source: Brain Pathol. 2023 Aug 24;34(1):e13202. doi: 10.1111/bpa.13202 (PMC10711261; doi:10.1111/bpa.13202)

***Supplementary data 1***

**Lipidomics sample collection and LC-MS/MS Analysis**

**Plasma sample collection**

After 8–12 h of fasting, venous blood (6 ml) was drawn from the anterior elbow vein using EDTA vacuum blood sampling tubes (BD, Franklin Lakes, NJ, USA). At 4°C, the samples were centrifuged at 3000 rpm for 10 min (Allegra X-15R; Beckman Coulter, USA). The supernatant plasma was pipetted into sterile polypropylene cryotubes and stored in a refrigerator at −80°C.

**Sample Preparation**

Lipids were extracted using the MTBE method for sample preparation and lipid extraction. Brieﬂy, Samples (100 μL) were accurately measured and spiked with appropriate amounts of internal lipid standards, then homogenized with 100 μL water and 240 μL methanol. After that, 800 μL of MTBE was added, and the mixture was treated with ultrasound for 20 min at 4 °C followed by sitting still for 30 min at room temperature. The solution was centrifuged at 14,000× g for 15 min at 10 °C, and the upper layer was obtained and dried under nitrogen. The lipid extracts were re-dissolved in 200 μL of 90% isopropanol/acetonitrile, centrifuged at 14,000× g for 15 min, and ﬁnally 3 μL of the sample was injected into Nexera UHPLC LC-30A system (Shimadzu, Japan). 10 μL of each sample was collected to prepare quality control (QC) samples.

**Mass Spectrometry**

CSH C18 column (1.7 μm, 2.1 mm × 100 mm, Waters) was used to separate the lipids.. A solution of 200 μL of 90%isopropanol/acetonitrile was added to the lipid extracts. Centrifuged at 14,000 × g for 15 min, and ﬁnally 3 μL of the sample was injected. Mobile phase composition A: acetonitrile-water (6:4, v/v); B: acetonitrile-isopropanol (1:9, v/v), both containing 0.1% formic acid and 0.1 mM ammonium formate. The gradient elution program was as follows: 0 - 2 min, B was maintained at 30%; 2 - 25 min, B changed linearly from 30% to 100%; 25 - 35 min, B was maintained at 30%. The electrospray ionization (ESI) positive and negative ion modes were used for the detection, respectively. Mass spectrometry analysis was performed using a Q Exactive mass spectrometer. ESI (Electron Spray Ionization) parameters: source temperature, 300 °C; capillary temperature, 350 °C; spray voltage,3000 V; S-Lens RF Level, 50%; scan ranges, 200–1800 m/z. The mass charge ratio of lipid molecules and lipid fragments was collected using the following methods: 10 fragments (MS2scan, HCD) were collected after each full scan. The resolution of MS1 is 70, 000 at m/z 200, and that of MS2 is 17, 500 at m/z 200.

The peaks of lipid molecules and internal standard lipid molecules were extracted and identified using Lipidsearch (Thermo Fisher Scientific, USA). The main parameters were: 5ppm for precursor tolerance, 5ppm for product tolerance, and 5% for production threshold.

***Supplementary data 2***

**Lipid metabolomics data processing**

Raw lipid metabolism data were processed using LipidSearch software version 4.2 (Thermo Scientific™) with peak alignment, peak extraction, retention time correction, and removal of ion peaks with > 50% missing values within each group. The processed data were imported into SIMPCA-P 16.1 (Umetrics, Umea, Sweden) for orthogonal partial least squares discriminant analysis (OPLS-DA), and the variable important in projection (VIP) score was obtained. Student’s t-tests and fold change (FC) were analyzed using PASS 16.

***Supplementary data 3***

**Lipid metabolomics sample size calculation:**

There are many lipid metabolites, and it is impossible for us to calculate the sample size according to each lipid. We select representative PE for sample size calculation. Based on the results of similar published literature, the mean PE values for the AD, MCI and NC groups were expected to be 7.04, 4.76, and 6.85, respectively, with a standard deviation of 2.65. With a degree of certainty of 1-β=0.80 and a test level of α=0.05, the sample sizes of the three groups were equal and sample size estimation was performed using the PASS 2021 software according to the following formula.

References: Peña-Bautista C, Álvarez-Sánchez L, Roca M, García-Vallés L, Baquero M, Cháfer-Pericás C. Plasma Lipidomics Approach in Early and Specific Alzheimer's Disease Diagnosis. J Clin Med. 2022 Aug 27;11(17):5030. doi: 10.3390/jcm11175030. PMID: 36078960; PMCID: PMC9457360.

$$n=\Psi^{2}\left[ \sum_{i=1}^{k} {s_{i}}^{2}/k \right]/\left[ \sum_{i=1}^{k} \left( \bar{X_{i}}-\bar{X} \right)^{2}/(k-1) \right]$$

The minimum required sample size for all three groups was 23 cases, based on pre-set parameters, calculated using the One-Way Analysis of Variance F-Tests under the Means menu of the PASS 2021 software.


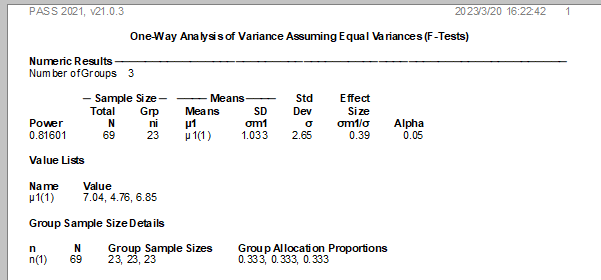


**Sample size calculation for oxidative stress indicators:**

According to the results of pre-experiment, the mean MDA values of AD group, aMCI group and NC group were 2.54, 2.21, and 1.78, respectively, and the standard deviation was 0.67. Power 1-β=0.90, test level α=0.05, AD group: aMCI group: NC group =1:1:1. Sample size estimation was performed using PASS 2021 software according to the following formula:

$$n=\Psi^{2}\left[ \sum_{i=1}^{k} {s_{i}}^{2}/k \right]/\left[ \sum_{i=1}^{k} \left( \bar{X_{i}}-\bar{X} \right)^{2}/(k-1) \right]$$

According to the preset parameters, MDA was calculated by One-Way Analysis of Variance F-Tests under the Means menu of PASS 2021 software, and the minimum required sample size of the three groups was 21 cases.


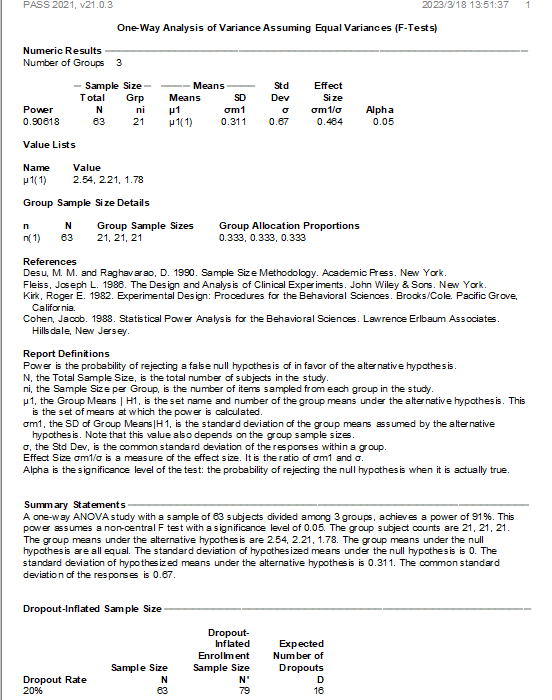

Supplement: Supplementary file 1 — Supplementary Data 1. Lipidomics sample collection and LC‐MS/MS analysis. Supplementary Data 2. Lipid metabolomics data processing. Supplementary Data 3. Lipid metabolomics sample size calculation. [file BPA-34-e13202-s001.docx]
