# Supplementary material for: Lipid metabolism and oxidative stress in patients with Alzheimer's disease and amnestic mild cognitive impairment
Source: Brain Pathol. 2023 Aug 24;34(1):e13202. doi: 10.1111/bpa.13202 (PMC10711261; doi:10.1111/bpa.13202)
Supplement: Supplementary file 2 — Supplementary Figure S1. The horizontal coordinates represent all experimental and QC samples, the vertical coordinates reflect confidence intervals, and the red line defines the 99% confidence interval range. Supplementary Figure S2. The horizontal coordinates of the graph indicate the retention time of each peak and the vertical coordinates indicate the intensity values of the peaks. QC sample experimental results showed that the chromatographic peak response intensity and retention time of QC samples basically overlapped, indicating that the experimental repeatability was good. (A) Base Peak chromatogram (BPC) overlapping of all QC samples in negative ion mode. (B) Base Peak chromatogram (BPC) overlapping of all QC samples in positive ion mode. Pearson correlation analysis was performed on the QC samples. A general correlation coefficient greater than 0.9 indicates a good correlation. The experimental results showed that the correlation coefficients between QC samples were all above 0.9, indicating that the experimental repeatability was excellent. Supplementary Figure S3. The abscissa and ordinate in the figure represent each QC sample. The points in each cell represent the ion peaks (metabolites) extracted from the QC samples, and the abscissa and ordinate represent the log values of the ion peak signal intensity values. Multivariate Control Chart (MCC) is a multivariate statistical model established based on the ion peaks detected in QC samples, and is a quality management tool used to monitor and judge whether the instrument status is stable. Each point in the multivariate control chart represents a QC sample, and the X‐axis is the order in which all QC samples were loaded. The points in the graph fluctuate up and down because of fluctuations in the state of the instrument. Generally, the normal range is within plus or minus 3 standard deviations. The experimental results showed that the fluctuation of QC samples was within the range of plus or minus 3 standa [file BPA-34-e13202-s002.docx]

Supplementary Figure S1

The Hotelling's T2 test tests samples by multivariate modelling, defining 95% or 99% confidence intervals, and can be used to diagnose outlier samples, with samples outside the red line generally being serious outliers. The results of Hotelling's T2 test in this study showed that the LOAD3 sample was outside the 99% confidence interval.


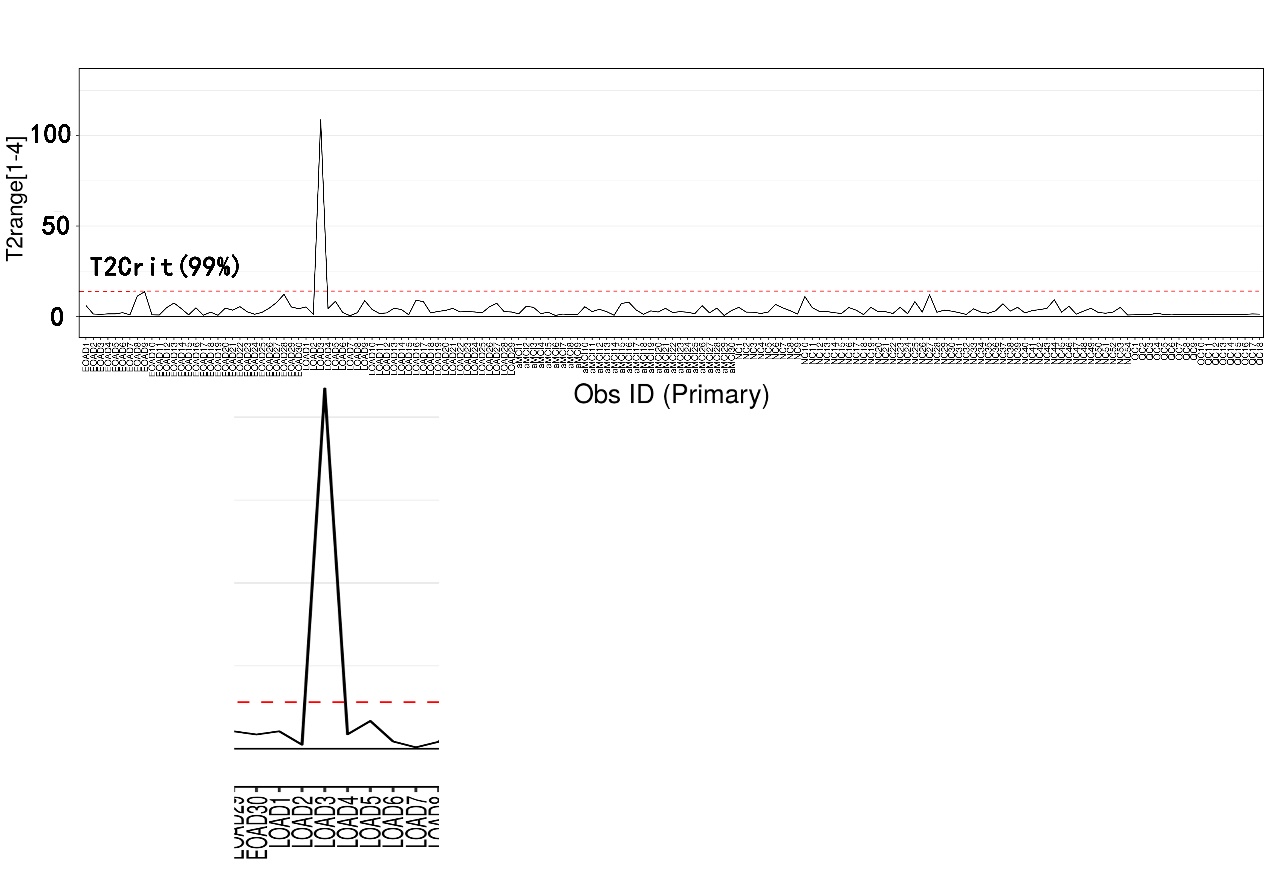


Supplementary Figure 1: The horizontal coordinates represent all experimental and QC samples, the vertical coordinates reflect confidence intervals, and the red line defines the 99% confidence interval range.

Supplementary Figure S2


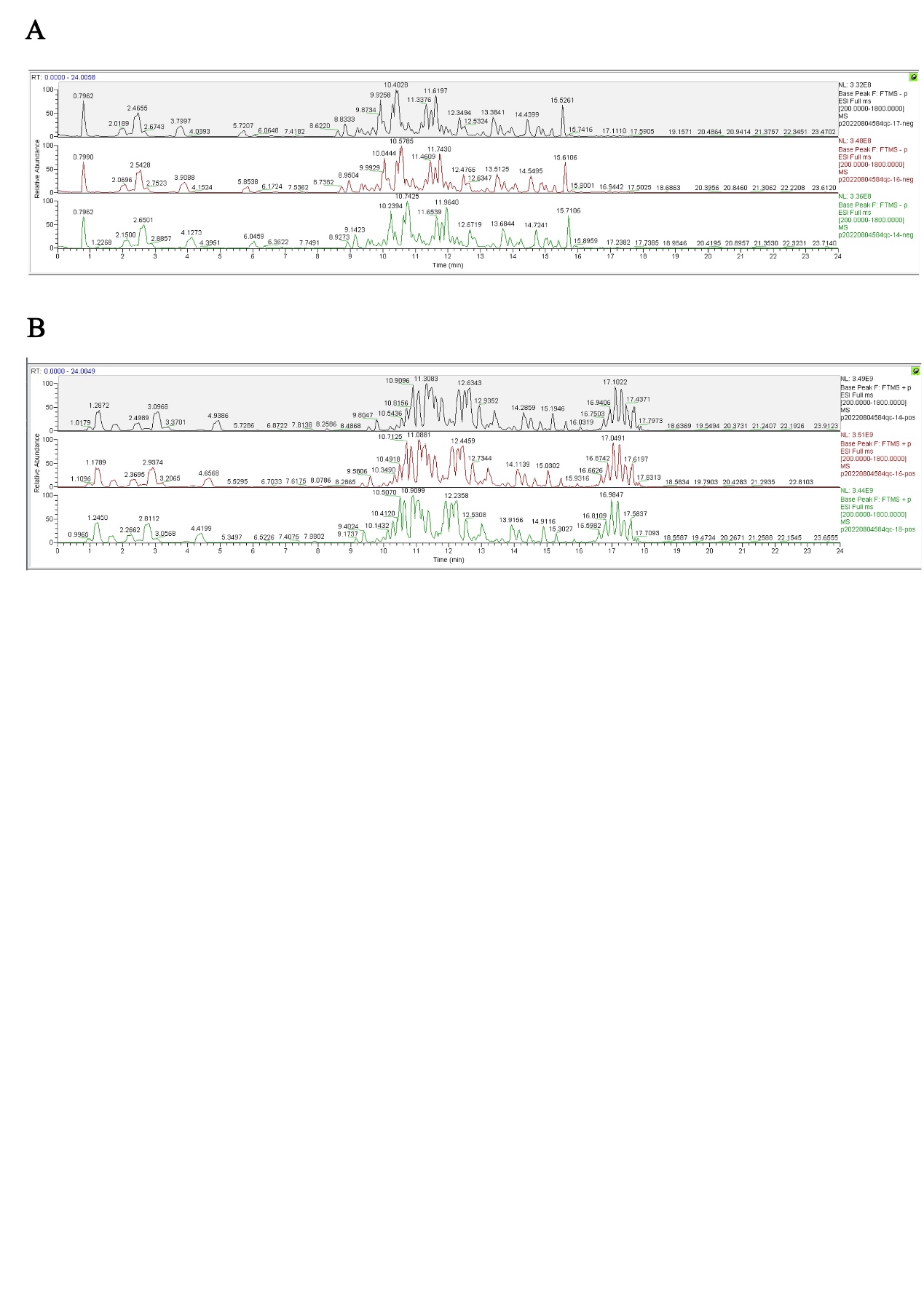


Supplementary Figure S2. The horizontal coordinates of the graph indicate the retention time of each peak and the vertical coordinates indicate the intensity values of the peaks. QC sample experimental results showed that the chromatographic peak response intensity and retention time of QC samples basically overlapped, indicating that the experimental repeatability was good. (A) Base Peak chromatogram (BPC) overlapping of all QC samples in negative ion mode. (B) Base Peak chromatogram (BPC) overlapping of all QC samples in positive ion mode.

Supplementary Figure S3

Pearson correlation analysis was performed on the QC samples. A general correlation coefficient greater than 0.9 indicates a good correlation. The experimental results showed that the correlation coefficients between QC samples were all above 0.9, indicating that the experimental repeatability was excellent.


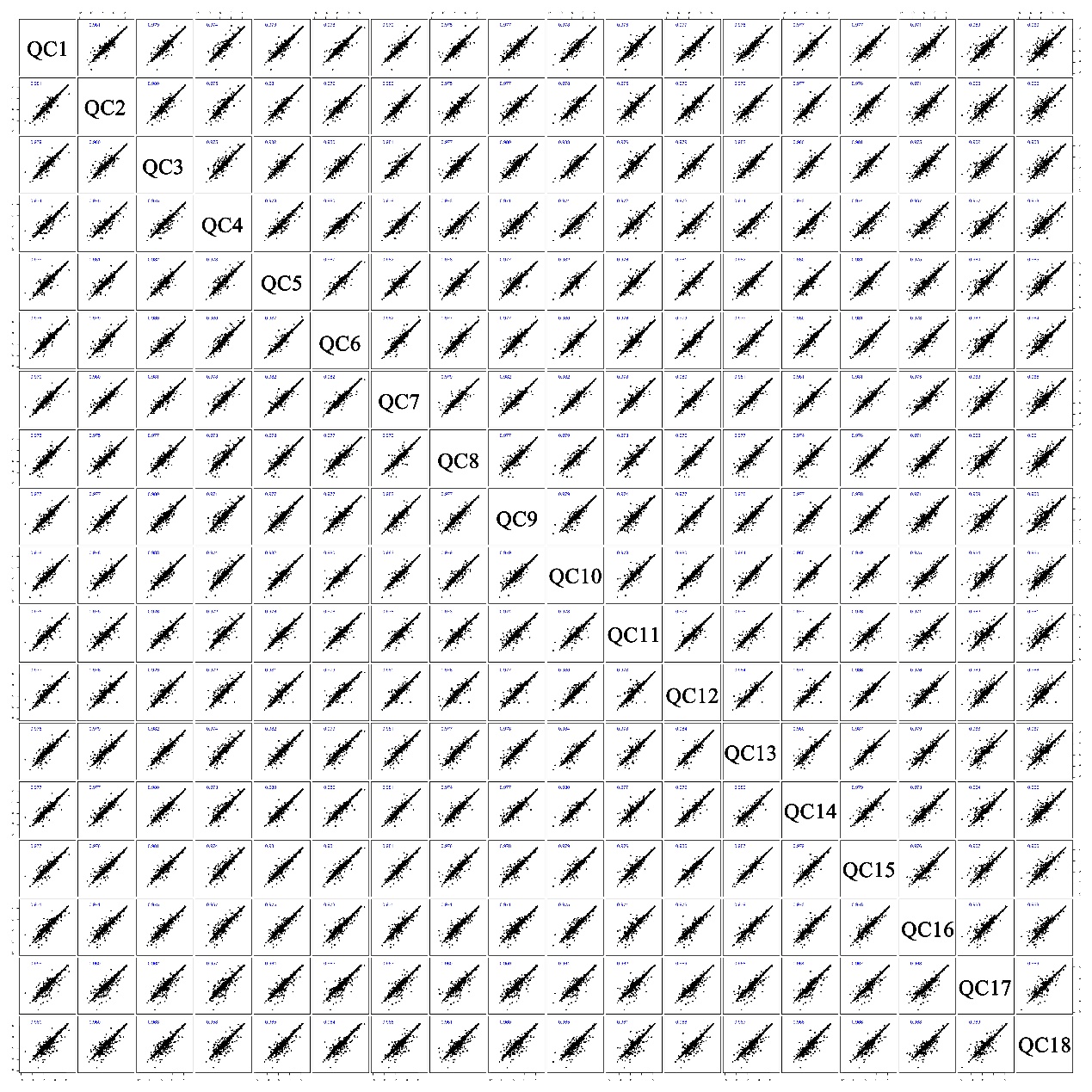


Supplementary Figure S3.The abscissa and ordinate in the figure represent each QC sample. The points in each cell represent the ion peaks (metabolites) extracted from the QC samples, and the abscissa and ordinate represent the log values of the ion peak signal intensity values.

Supplementary Figure S4

Multivariate Control Chart (MCC) is a multivariate statistical model established based on the ion peaks detected in QC samples, and is a quality management tool used to monitor and judge whether the instrument status is stable. Each point in the multivariate control chart represents a QC sample, and the X-axis is the order in which all QC samples were loaded. The points in the graph fluctuate up and down due to fluctuations in the state of the instrument. Generally, the normal range is within plus or minus 3 standard deviations. The experimental results showed that the fluctuation of QC samples was within the range of plus or minus 3 standard deviations, reflecting that the fluctuation of the instrument was within the normal range, and the data could be used for subsequent analysis.


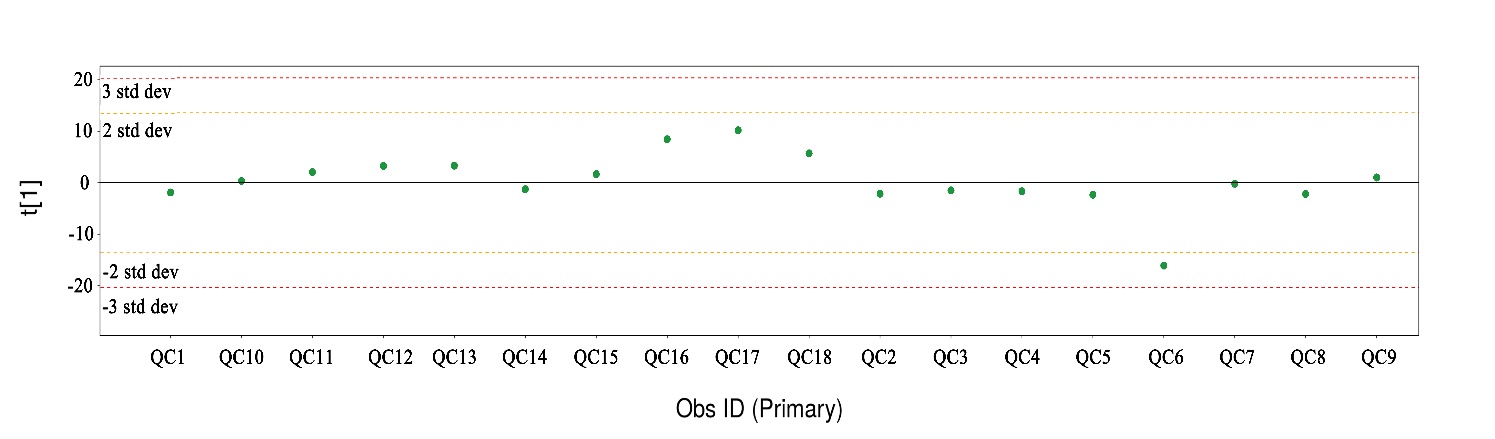


Supplementary Figure S4. The abscissa represents each QC sample, the ordinate reflects the standard deviation, and the yellow and red lines define a range of plus or minus 2 and 3 standard deviations, respectively.

Supplementary Figure S5


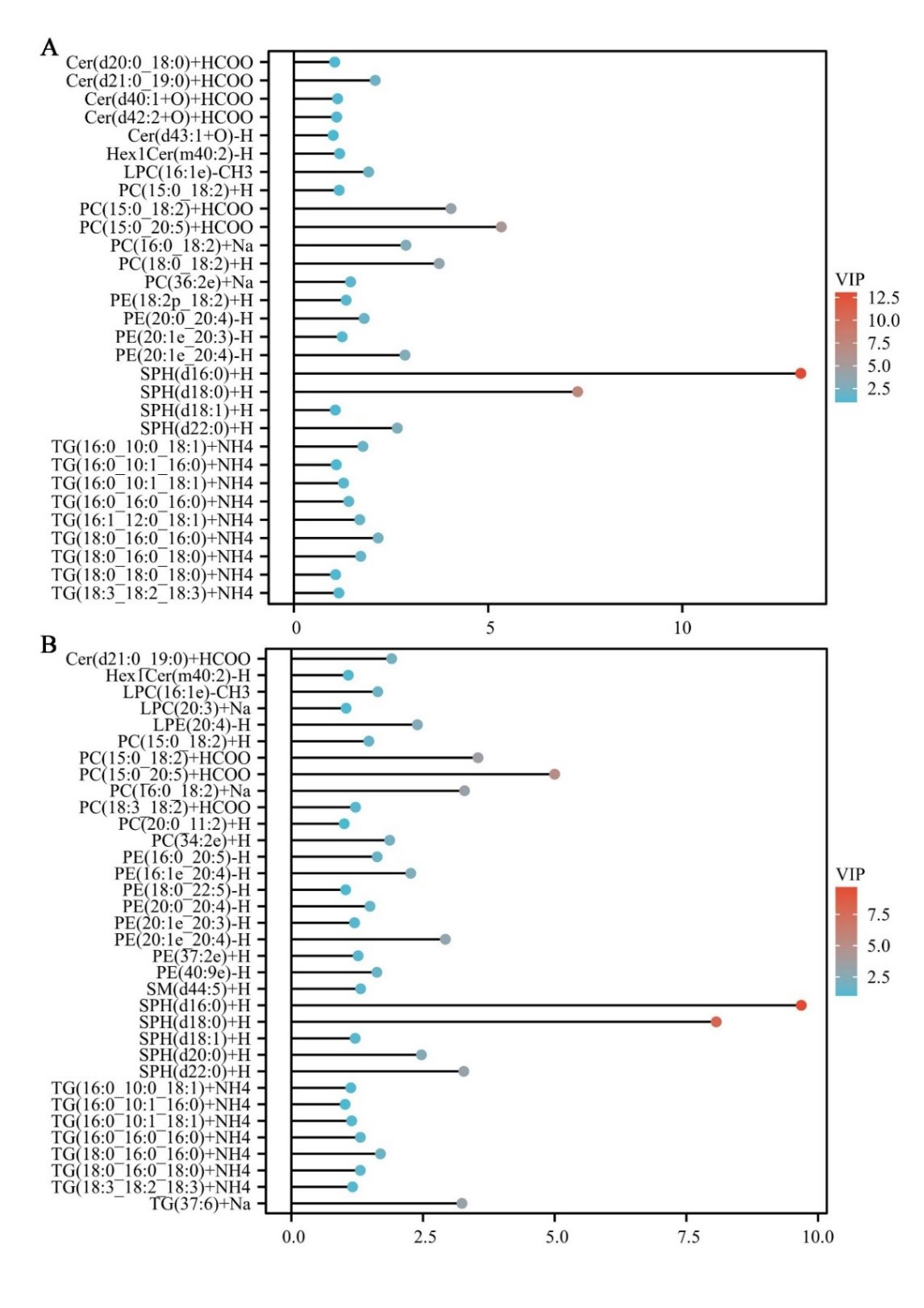


Supplementary Figure S5. (A) The variable important in projection (VIP) score of AD and NC. (B) VIP score of aMCI and NC.
