# Supplementary material for: Lipid metabolism and oxidative stress in patients with Alzheimer's disease and amnestic mild cognitive impairment
Source: Brain Pathol. 2023 Aug 24;34(1):e13202. doi: 10.1111/bpa.13202 (PMC10711261; doi:10.1111/bpa.13202)
Supplement: Supplementary file 3 — Supplementary Table S1. Evaluation parameters of OPLS‐DA model. Supplementary Table S2. Lipid metabolites altered in plasma of AD versus NC. Supplementary Table S3. Lipid metabolites altered in plasma of AD versus NC (analysis of covariance). Supplementary Table S4. Lipid metabolites altered in plasma of aMCI versus NC. Supplementary Table S5. Lipid metabolites altered in plasma of aMCI versus NC (analysis of covariance). Supplementary Table S6. Identification of potential markers for AD (adjusted for none). Supplementary Table S7. Identification of potential markers for AD (adjusted for age, gender, education level). Supplementary Table S8. ROC analysis of SPH(d16:0) + H, SPH(d18:1) + H, and SPH(d18:0) + H in the diagnosis of AD. Supplementary Table S9. Identification of potential markers for aMCI (adjusted for none). Supplementary Table S10. Identification of potential markers for aMCI (adjusted for age, gender, education level). Supplementary Table S11. ROC analysis of SPH(d16:0) + H, SPH(d18:1) + H, and SPH(d18:0) + H in the diagnosis of aMCI. Supplementary Table S12. Correlation between lipid metabolites and oxidative stress. Supplementary Table S13. Correlation between lipid metabolites and oxidative stress. [file BPA-34-e13202-s003.docx]

Supplementary Table S1

Evaluation parameters of OPLS-DA model

| Type | R^2^X(cum) | R^2^Y(cum) | Q^2^(cum) | Group |
| --- | --- | --- | --- | --- |
| OPLS-DA | 0.483 | 0.906 | 0.745 | AD vs NC |
| OPLS-DA | 0.58 | 0.976 | 0.778 | aMCI vs NC |
| OPLS-DA | 0.407 | 0.306 | -0.234 | AD vs aMCI |

Evaluation parameters of the OPLS-DA model: generally, Q^2^ is more significant than 0.5, indicating a stable and reliable model, 0.3 < Q^2^ ≤ 0.5, indicating good model stability, and Q^2^ < 0.3, indicating low model reliability.

Supplementary Table S2.

Lipid metabolites altered in plasma of AD vs. NC.

| LipidIon | CalMz | RT-(min) | FC | P-value | VIP |
| --- | --- | --- | --- | --- | --- |
| LPC(18:0)+HCOO | 568.362 | 4.352111 | 1.217978 | 0.032555 | 6.431327 |
| Cer(d18:1_16:0)+HCOO | 582.5103 | 11.71174 | 1.174642 | 0.000133 | 1.457822 |
| LPC(20:4)+HCOO | 588.3307 | 2.004093 | 0.751601 | 0.001443 | 1.7896 |
| Cer(m22:1_16:0+O)+HCOO | 638.5729 | 14.294 | 0.785254 | 0.000501 | 1.688052 |
| Cer(d20:0_18:0)+HCOO | 640.5885 | 14.63623 | 0.525561 | 7.63E-06 | 1.048808 |
| Cer(d16:1_24:1)+HCOO | 664.5885 | 14.13824 | 0.820337 | 0.002237 | 1.767911 |
| Cer(d18:1_22:0)+HCOO | 666.6042 | 15.00735 | 0.853587 | 0.012729 | 2.373565 |
| Cer(d21:0_19:0)+HCOO | 668.6198 | 15.6201 | 0.597532 | 6.44E-07 | 2.092554 |
| Cer(t17:0_22:0)+HCOO | 670.5991 | 15.4798 | 1.101935 | 0.045924 | 1.036199 |
| Cer(d43:1+O)-H | 678.6406 | 15.834 | 0.629717 | 1.4E-05 | 1.009927 |
| Cer(d40:1+O)+HCOO | 682.5991 | 14.57258 | 0.655916 | 3.14E-06 | 1.12286 |
| Cer(t18:0_22:0)+HCOO | 684.6148 | 14.43555 | 0.812692 | 0.004597 | 1.017596 |
| Cer(d18:1_24:0)+HCOO | 694.6355 | 15.58488 | 0.878899 | 0.040877 | 3.350199 |
| PE(16:1e_18:1)-H | 700.5287 | 12.74026 | 0.761474 | 5.57E-06 | 1.25332 |
| Cer(d42:2+O)+HCOO | 708.6148 | 14.84649 | 0.647623 | 2.08E-05 | 1.099362 |
| Cer(t18:1_24:0)+HCOO | 710.6304 | 15.69238 | 0.73084 | 0.002218 | 1.109052 |
| PE(16:1_18:2)-H | 712.4923 | 10.20895 | 1.431023 | 0.017016 | 1.228281 |
| PE(16:0_18:2)-H | 714.5079 | 11.425 | 1.243966 | 0.007279 | 2.280992 |
| PE(16:1e_20:4)-H | 722.513 | 11.64867 | 0.709514 | 1.34E-08 | 6.052095 |
| PE(18:1e_18:1)-H | 728.56 | 13.57125 | 0.772097 | 0.000285 | 1.634362 |
| PE(18:0_18:3)-H | 740.5236 | 11.82 | 1.471233 | 0.011745 | 1.257145 |
| PC(16:1_18:2)-CH3 | 740.5236 | 11.46065 | 1.447727 | 0.000201 | 2.173507 |
| PE(18:1_18:1)-H | 742.5392 | 12.32712 | 1.177641 | 0.034919 | 2.028358 |
| PE(16:1e_22:6)-H | 746.513 | 11.3315 | 0.796162 | 0.000432 | 2.623774 |
| PE(18:2e_20:4)-H | 748.5287 | 11.73066 | 0.877331 | 0.019757 | 1.568181 |
| PE(18:1e_20:3)-H | 752.56 | 13.27221 | 0.787157 | 0.005416 | 1.125566 |
| PE(18:0_20:5)-H | 764.5236 | 11.27146 | 0.763611 | 0.002029 | 2.160589 |
| Hex1Cer(m40:2)-H | 764.641 | 15.901 | 0.525251 | 8.38E-13 | 1.175334 |
| PC(18:2_18:2)-CH3 | 766.5392 | 12.30632 | 0.751753 | 1.46E-07 | 4.308781 |
| CL(78:5)-2H | 768.5493 | 12.633 | 0.762442 | 1.67E-07 | 1.423147 |
| PE(18:1e_22:5)-H | 776.56 | 13.22334 | 0.764203 | 0.002304 | 1.491813 |
| PE(20:1e_20:4)-H | 778.5756 | 13.88565 | 0.591078 | 4.45E-11 | 2.859644 |
| PE(20:1e_20:3)-H | 780.5913 | 13.89113 | 0.577128 | 7.47E-11 | 1.241756 |
| PE(18:2_22:6)-H | 786.5079 | 10.202 | 0.670019 | 0.011075 | 1.074793 |
| PC(15:0_18:2)+HCOO | 788.5447 | 11.95331 | 0.083971 | 1.56E-13 | 4.043282 |
| PE(20:0_20:4)-H | 794.5705 | 13.3279 | 0.435941 | 3.66E-13 | 1.806902 |
| PC(16:1_18:2)+HCOO | 800.5447 | 9.977385 | 1.161765 | 0.007376 | 2.334807 |
| PC(18:0_16:0)+HCOO | 806.5917 | 12.56977 | 0.84524 | 0.000108 | 1.090097 |
| PC(15:0_20:5)+HCOO | 810.5291 | 10.912 | 0.110009 | 8.95E-15 | 5.338657 |
| PC(18:3_18:2)+HCOO | 824.5447 | 9.543228 | 1.369891 | 0.002312 | 1.23507 |
| SM(d40:1)+HCOO | 831.6597 | 14.11191 | 0.875698 | 0.007352 | 2.942303 |
| PC(18:0_18:1)+HCOO | 832.6073 | 12.67182 | 0.906715 | 0.036751 | 1.229346 |
| PC(16:1e_22:0)+HCOO | 846.6593 | 15.02376 | 1.201825 | 7.94E-05 | 1.098785 |
| PC(18:0_20:4)+HCOO | 854.5917 | 11.94536 | 0.87831 | 0.009491 | 2.347821 |
| PC(18:0_20:3)+HCOO | 856.6073 | 12.75729 | 0.720422 | 0.000561 | 1.253127 |
| Hex1Cer(d18:1_24:0)+HCOO | 856.6883 | 14.94446 | 1.167796 | 0.01066 | 2.134766 |
| SM(d42:2)+HCOO | 857.6753 | 14.01288 | 0.875853 | 0.025944 | 2.348157 |
| SM(d42:1)+HCOO | 859.691 | 14.62909 | 0.890904 | 0.0126 | 2.546006 |
| Hex1Cer(t18:0_24:1)+HCOO | 872.6832 | 15.08114 | 0.757174 | 0.00182 | 1.373886 |
| PI(18:0_20:4)-H | 885.5499 | 10.90747 | 0.897565 | 0.025617 | 2.598908 |
| SPH(d18:1)+H | 300.2897 | 3.138358 | 2.752819 | 1.08E-10 | 1.063983 |
| SPH(d18:0)+H | 302.3054 | 2.827902 | 2.882602 | 3.59E-12 | 7.304335 |
| SPH(d20:0)+H | 330.3367 | 4.755926 | 1.540415 | 0.036597 | 1.728992 |
| ChE(20:4)+NH4 | 690.6184 | 17.64742 | 0.750747 | 0.025717 | 1.222484 |
| SPH(d16:0)+H | 274.2741 | 1.776995 | 2.227576 | 1.66E-28 | 13.05118 |
| PE(18:2p_18:2)+H | 724.5276 | 14.21156 | 0.636184 | 0.000113 | 1.343947 |
| TG(16:0_10:1_16:0)+NH4 | 738.6606 | 17.14933 | 10.45202 | 0.003428 | 1.089623 |
| PC(15:0_18:2)+H | 744.5538 | 14.61232 | 1.703917 | 0.001384 | 1.163301 |
| SPH(d22:0)+H | 358.368 | 6.495785 | 2.388232 | 9.9E-07 | 2.660929 |
| TG(16:0_10:1_18:1)+NH4 | 764.6763 | 17.17512 | 5.292064 | 0.003468 | 1.276998 |
| TG(16:0_10:0_18:1)+NH4 | 766.6919 | 17.34897 | 4.990619 | 0.000918 | 1.774843 |
| PC(16:0_18:2)+Na | 780.5514 | 12.75761 | 1.640565 | 0.018242 | 2.882607 |
| PC(18:0_18:2)+H | 786.6007 | 14.3003 | 0.164267 | 0.041323 | 3.738801 |
| TG(16:1_12:0_18:1)+NH4 | 792.7076 | 17.38254 | 2.232056 | 0.003027 | 1.693652 |
| PC(36:2e)+Na | 794.6034 | 14.03722 | 0.424417 | 0.007281 | 1.457024 |
| TG(18:1_12:0_18:2)+NH4 | 818.7232 | 17.40195 | 1.425475 | 0.018352 | 1.472144 |
| TG(16:0_16:0_16:0)+NH4 | 824.7702 | 17.61433 | 1.909018 | 4.43E-05 | 1.410212 |
| TG(18:0_16:0_16:0)+NH4 | 852.8015 | 17.74533 | 1.863226 | 0.000201 | 2.166382 |
| TG(16:1_18:2_18:3)+NH4 | 868.7389 | 17.31108 | 1.279922 | 0.043905 | 1.243397 |
| TG(18:0_16:0_18:0)+NH4 | 880.8328 | 17.93874 | 1.980941 | 7.57E-05 | 1.718866 |
| TG(18:3_18:2_18:3)+NH4 | 892.7389 | 17.20639 | 1.595173 | 0.020661 | 1.155149 |
| TG(18:3_18:2_18:2)+NH4 | 894.7545 | 17.33151 | 1.387737 | 0.002553 | 2.91439 |
| TG(18:1_18:2_18:3)+NH4 | 896.7702 | 17.48404 | 1.165047 | 0.0472 | 2.944176 |
| TG(18:1_18:2_18:2)+NH4 | 898.7858 | 17.61224 | 1.130994 | 0.014465 | 3.20415 |
| TG(18:0_18:0_18:0)+NH4 | 908.8641 | 18.01592 | 1.708819 | 0.000483 | 1.071954 |
| TG(18:1_18:2_22:6)+NH4 | 946.7858 | 17.42778 | 0.812125 | 0.022239 | 1.575258 |
| LPE(16:0)-H | 452.2783 | 2.626173 | 1.35904 | 0.03586 | 1.032158 |
| LPC(16:1e)-CH3 | 464.3147 | 5.143325 | 0.494851 | 3.83E-15 | 1.921194 |
| LPE(18:2)-H | 476.2783 | 2.115488 | 1.236467 | 0.028051 | 1.49914 |

Supplementary Table S3.

Lipid metabolites altered in plasma of AD vs. NC(Analysis of covariance).

| LipidIon | ANCOVA_pvalue | ANCOVA_pvalue |
| --- | --- | --- |
|  |  | (Remove outliers) |
| Cer(d18:1_24:0)+HCOO | 0.140996057 | 0.140703026 |
| TG(16:1_18:2_18:3)+NH4 | 0.096298222 | 0.108254953 |
| TG(18:1_18:2_18:3)+NH4 | 0.090099716 | 0.103594809 |
| SM(d42:2)+HCOO | 0.074292148 | 0.073471531 |
| LPE(18:2)-H | 0.066436151 | 0.07186471 |
| Cer(d18:1_22:0)+HCOO | 0.063771258 | 0.070187554 |
| SPH(d20:0)+H | 0.057652151 | 0.069675792 |
| TG(18:1_12:0_18:2)+NH4 | 0.062370488 | 0.065620552 |
| SM(d42:1)+HCOO | 0.057963144 | 0.058148305 |
| TG(18:1_18:2_22:6)+NH4 | 0.065096685 | 0.05583723 |
| PI(18:0_20:4)-H | 0.047782659 | 0.054360649 |
| PE(18:2e_20:4)-H | 0.033832372 | 0.043441296 |
| PC(18:0_20:4)+HCOO | 0.039853512 | 0.041577968 |
| TG(18:3_18:2_18:3)+NH4 | 0.033549697 | 0.038639001 |
| ChE(20:4)+NH4 | 0.041003687 | 0.037701455 |
| SM(d40:1)+HCOO | 0.038068723 | 0.034688474 |
| LPE(16:0)-H | 0.035194299 | 0.034417041 |
| TG(18:1_18:2_18:2)+NH4 | 0.027871649 | 0.0306965 |
| PE(18:1_18:1)-H | 0.024818165 | 0.023584995 |
| PC(18:0_18:1)+HCOO | 0.022784008 | 0.023060286 |
| LPC(18:0)+HCOO | 0.031059623 | 0.021834939 |
| PE(16:1_18:2)-H | 0.023725769 | 0.021440983 |
| PC(18:0_18:2)+H | 0.020089494 | 0.021177945 |
| Hex1Cer(d18:1_24:0)+HCOO | 0.020888778 | 0.020496941 |
| TG(16:1_12:0_18:1)+NH4 | 0.020097729 | 0.018633358 |
| Cer(d16:1_24:1)+HCOO | 0.016947819 | 0.018532428 |
| PC(18:3_18:2)+HCOO | 0.017893919 | 0.017965202 |
| Cer(t17:0_22:0)+HCOO | 0.01311327 | 0.017048145 |
| PE(18:2_22:6)-H | 0.013687649 | 0.014122196 |
| PE(18:1e_22:5)-H | 0.018671069 | 0.013955957 |
| PC(16:0_18:2)+Na | 0.011377198 | 0.01336178 |
| TG(16:0_10:1_18:1)+NH4 | 0.013759192 | 0.013098326 |
| Cer(t18:1_24:0)+HCOO | 0.018091553 | 0.012727705 |
| Hex1Cer(t18:0_24:1)+HCOO | 0.011401957 | 0.012115629 |
| TG(16:0_10:1_16:0)+NH4 | 0.012655081 | 0.011709029 |
| PE(18:1e_20:3)-H | 0.010525156 | 0.010827017 |
| Cer(t18:0_22:0)+HCOO | 0.010638683 | 0.010066057 |
| PE(18:0_18:3)-H | 0.010246628 | 0.009516311 |
| Cer(m22:1_16:0+O)+HCOO | 0.006994933 | 0.007856908 |
| PC(16:1_18:2)+HCOO | 0.006219925 | 0.007624342 |
| PE(16:1e_22:6)-H | 0.007439596 | 0.007231823 |
| TG(16:0_10:0_18:1)+NH4 | 0.007551241 | 0.006618376 |
| PE(18:0_20:5)-H | 0.0042661 | 0.00517854 |
| PC(36:2e)+Na | 0.003893967 | 0.004384388 |
| TG(18:3_18:2_18:2)+NH4 | 0.003382622 | 0.00418705 |
| TG(18:0_18:0_18:0)+NH4 | 0.004840131 | 0.00371096 |
| TG(18:0_16:0_16:0)+NH4 | 0.002828203 | 0.003127634 |
| PC(18:0_20:3)+HCOO | 0.002908129 | 0.003058694 |
| PE(16:0_18:2)-H | 0.003044869 | 0.002789772 |
| PC(15:0_18:2)+H | 0.00120136 | 0.001338846 |
| LPC(20:4)+HCOO | 0.001759013 | 0.001208365 |
| PE(18:1e_18:1)-H | 0.000864038 | 0.001014943 |
| PE(18:2p_18:2)+H | 0.000515287 | 0.000621504 |
| TG(18:0_16:0_18:0)+NH4 | 0.000494559 | 0.000583923 |
| TG(16:0_16:0_16:0)+NH4 | 0.000477083 | 0.000549775 |
| PC(16:1e_22:0)+HCOO | 0.000437975 | 0.000535287 |
| Cer(d42:2+O)+HCOO | 0.000357363 | 0.000287087 |
| PC(16:1_18:2)-CH3 | 0.000293608 | 0.000245962 |
| PC(18:0_16:0)+HCOO | 0.000184955 | 0.000231853 |
| Cer(d43:1+O)-H | 0.000217098 | 0.000205333 |
| Cer(d20:0_18:0)+HCOO | 0.000218363 | 0.000174321 |
| PE(16:1e_18:1)-H | 4.90006E-05 | 6.50156E-05 |
| Cer(d18:1_16:0)+HCOO | 5.82535E-05 | 5.83494E-05 |
| Cer(d40:1+O)+HCOO | 5.6439E-05 | 5.3906E-05 |
| Cer(d21:0_19:0)+HCOO | 7.58132E-06 | 1.01109E-05 |
| PC(18:2_18:2)-CH3 | 5.78806E-06 | 7.00854E-06 |
| CL(78:5)-2H | 3.52792E-06 | 4.32801E-06 |
| SPH(d22:0)+H | 1.06506E-06 | 1.39242E-06 |
| PE(16:1e_20:4)-H | 4.74056E-07 | 6.11808E-07 |
| PE(20:1e_20:3)-H | 7.00469E-10 | 1.09901E-09 |
| PE(20:1e_20:4)-H | 5.24076E-10 | 7.88286E-10 |
| SPH(d18:1)+H | 2.05351E-10 | 1.26134E-10 |
| Hex1Cer(m40:2)-H | 5.38096E-11 | 7.48845E-11 |
| PE(20:0_20:4)-H | 1.6899E-11 | 1.64334E-11 |
| PC(15:0_18:2)+HCOO | 7.64116E-12 | 1.09269E-11 |
| PC(15:0_20:5)+HCOO | 2.02212E-12 | 3.02549E-12 |
| LPC(16:1e)-CH3 | 1.70398E-12 | 2.69439E-12 |
| SPH(d18:0)+H | 4.55523E-13 | 7.45895E-13 |
| SPH(d16:0)+H | 1.47161E-27 | 3.03501E-27 |

Adjusted for age, gender, education level

Supplementary Table S4.

Lipid metabolites altered in plasma of aMCI vs. NC.

| LipidIon | CalMz | RT-(min) | FC | P-value | VIP |
| --- | --- | --- | --- | --- | --- |
| LPE(20:4)-H | 500.2783 | 2.035163 | 0.63183 | 0.002214 | 2.392412 |
| LPC(18:1)+HCOO | 566.3463 | 2.761972 | 0.741222 | 0.004256 | 1.020957 |
| LPC(18:0)+HCOO | 568.362 | 4.352111 | 1.317756 | 0.007462 | 6.119142 |
| LPC(20:4)+HCOO | 588.3307 | 2.004093 | 0.72093 | 0.004708 | 1.73955 |
| Cer(m22:1_16:0+O)+HCOO | 638.5729 | 14.294 | 0.737919 | 7.91E-05 | 1.805795 |
| Cer(d16:1_24:1)+HCOO | 664.5885 | 14.13824 | 0.793107 | 0.000838 | 1.811821 |
| Cer(d18:1_22:0)+HCOO | 666.6042 | 15.00735 | 0.813204 | 0.001987 | 2.490697 |
| Cer(d21:0_19:0)+HCOO | 668.6198 | 15.6201 | 0.572761 | 1.22E-05 | 1.903765 |
| Cer(t17:0_22:0)+HCOO | 670.5991 | 15.4798 | 1.229477 | 2.46E-06 | 1.847348 |
| Cer(d18:1_24:0)+HCOO | 694.6355 | 15.58488 | 0.872149 | 0.032408 | 2.688401 |
| Cer(t17:0_24:0)+HCOO | 698.6304 | 15.99608 | 1.209825 | 0.009027 | 1.076985 |
| PE(16:1e_18:1)-H | 700.5287 | 12.74026 | 0.818246 | 0.002131 | 1.015698 |
| PE(16:0_18:2)-H | 714.5079 | 11.425 | 1.28339 | 0.001895 | 2.571844 |
| PE(16:1e_20:4)-H | 722.513 | 12.133 | 3.093903 | 0.009396 | 2.267057 |
| PE(18:1e_18:1)-H | 728.56 | 13.57125 | 0.837552 | 0.041498 | 1.165 |
| PE(16:0_20:5)-H | 736.4923 | 10.39916 | 1.863582 | 0.014459 | 1.629269 |
| PE(18:0_18:3)-H | 740.5236 | 11.82 | 1.353152 | 0.005312 | 1.007582 |
| PC(16:1_18:2)-CH3 | 740.5236 | 11.46065 | 1.440789 | 3.07E-05 | 2.057973 |
| PE(18:2e_20:4)-H | 748.5287 | 11.73066 | 0.831625 | 0.007908 | 2.381626 |
| PE(18:0_20:5)-H | 764.5236 | 11.27146 | 0.763801 | 0.011584 | 1.888945 |
| Hex1Cer(m40:2)-H | 764.641 | 15.901 | 0.499188 | 3.64E-09 | 1.081318 |
| PC(18:2_18:2)-CH3 | 766.5392 | 12.30632 | 0.779387 | 2.69E-05 | 3.503323 |
| CL(78:5)-2H | 768.5493 | 12.633 | 0.794498 | 3.93E-05 | 1.219909 |
| PE(40:9e)-H | 770.513 | 10.99 | 3.242664 | 0.034142 | 1.623473 |
| PE(20:1e_20:4)-H | 778.5756 | 13.88565 | 0.603794 | 1.11E-08 | 2.924243 |
| PE(20:1e_20:3)-H | 780.5913 | 13.89113 | 0.588913 | 6.09E-08 | 1.199765 |
| PC(15:0_18:2)+HCOO | 788.5447 | 11.95331 | 0.04331 | 3.56E-09 | 3.543647 |
| PE(18:0_22:5)-H | 792.5549 | 12.25296 | 0.654303 | 1.51E-05 | 1.030993 |
| PE(20:0_20:4)-H | 794.5705 | 13.3279 | 0.502959 | 5.12E-08 | 1.492634 |
| PC(16:0_18:3)+HCOO | 800.5447 | 10.386 | 1.342149 | 0.027064 | 1.104302 |
| PC(16:1_18:2)+HCOO | 800.5447 | 9.977385 | 1.194244 | 0.004532 | 2.054623 |
| PC(18:0_16:0)+HCOO | 806.5917 | 12.56977 | 0.836332 | 4.09E-05 | 1.042908 |
| PC(15:0_20:5)+HCOO | 810.5291 | 10.912 | 0.044752 | 6.07E-11 | 4.998582 |
| PC(18:3_18:2)+HCOO | 824.5447 | 9.543228 | 1.550247 | 0.000957 | 1.218896 |
| SM(d40:1)+HCOO | 831.6597 | 14.11191 | 0.877971 | 0.005727 | 2.476916 |
| Hex1Cer(d18:1_23:0)+HCOO | 842.6727 | 14.77312 | 1.201954 | 0.009573 | 1.297697 |
| PC(16:1e_22:0)+HCOO | 846.6593 | 15.02376 | 1.268725 | 2.25E-05 | 1.108704 |
| PC(18:2_20:4)+HCOO | 850.5604 | 9.971575 | 0.876744 | 0.015414 | 1.183664 |
| Hex1Cer(d18:1_24:0)+HCOO | 856.6883 | 14.94446 | 1.225998 | 0.003843 | 2.205209 |
| PI(18:0_20:4)-H | 885.5499 | 10.90747 | 0.896522 | 0.024505 | 3.230729 |
| SPH(d18:1)+H | 300.2897 | 3.138358 | 3.42128 | 2.07E-15 | 1.212238 |
| SPH(d18:0)+H | 302.3054 | 2.827902 | 3.543251 | 2.31E-17 | 8.066728 |
| LPC(20:4)+H | 544.3398 | 3.977522 | 0.700177 | 0.029178 | 1.446895 |
| LPC(20:3)+Na | 568.3374 | 3.842937 | 2.076809 | 0.031209 | 1.039508 |
| SPH(d20:0)+H | 330.3367 | 4.755926 | 2.001025 | 0.00118 | 2.46938 |
| TG(37:6)+Na | 663.4595 | 15.008 | 1.893847 | 0.008419 | 3.238824 |
| SM(d16:1_16:0)+H | 675.5436 | 11.45287 | 1.35716 | 0.049682 | 1.236043 |
| SPH(d16:0)+H | 274.2741 | 1.776995 | 2.23283 | 1.21E-15 | 9.681395 |
| PC(20:0_11:2)+H | 716.5225 | 13.913 | 1.647474 | 1.01E-05 | 1.003882 |
| PC(32:0e)+H | 720.5902 | 14.61771 | 1.451505 | 0.00812 | 1.15002 |
| PE(18:2p_18:2)+H | 724.5276 | 14.21156 | 0.738143 | 0.027331 | 1.052131 |
| TG(16:0_10:1_16:0)+NH4 | 738.6606 | 17.14933 | 8.13531 | 0.000326 | 1.021084 |
| PE(37:3e)+H | 742.5745 | 14.056 | 1.352936 | 0.044505 | 1.779212 |
| PC(15:0_18:2)+H | 744.5538 | 14.61232 | 1.876991 | 4.83E-05 | 1.470379 |
| PC(34:2e)+H | 744.5902 | 14.0603 | 1.720046 | 0.002727 | 1.866593 |
| PE(37:2e)+H | 744.5902 | 14.6966 | 1.567289 | 0.003391 | 1.267792 |
| PC(34:1e)+H | 746.6058 | 14.74543 | 1.45236 | 0.004879 | 1.419421 |
| SPH(d22:0)+H | 358.368 | 6.495785 | 2.876876 | 8.7E-10 | 3.274104 |
| PC(16:0_16:0)+Na | 756.5514 | 12.59317 | 1.498342 | 0.007812 | 2.023891 |
| PC(34:2)+H | 758.5694 | 13.41997 | 1.345139 | 0.032801 | 7.373343 |
| TG(16:0_10:1_18:1)+NH4 | 764.6763 | 17.17512 | 4.165659 | 5.65E-05 | 1.144723 |
| TG(16:0_10:0_18:1)+NH4 | 766.6919 | 17.34897 | 3.461673 | 0.000884 | 1.127997 |
| PC(16:0_18:2)+Na | 780.5514 | 12.75761 | 1.87773 | 0.004993 | 3.288274 |
| PC(38:5e)+H | 794.6058 | 14.28014 | 1.438393 | 0.018012 | 2.340408 |
| PC(38:4)+H | 810.6007 | 14.30035 | 1.425528 | 0.030673 | 5.240384 |
| SM(d18:1_24:2)+H | 811.6688 | 15.10531 | 1.35496 | 0.021675 | 2.535564 |
| TG(16:0_16:0_16:0)+NH4 | 824.7702 | 17.61433 | 2.066995 | 0.000135 | 1.310511 |
| PC(18:0_20:4)+Na | 832.5827 | 13.29338 | 1.418777 | 0.019649 | 1.753807 |
| SM(d44:5)+H | 835.6688 | 15.49945 | 0.392242 | 0.023635 | 1.314892 |
| TG(18:0_16:0_16:0)+NH4 | 852.8015 | 17.74533 | 1.872607 | 0.000881 | 1.690797 |
| TG(18:0_16:0_18:0)+NH4 | 880.8328 | 17.93874 | 1.909646 | 0.000707 | 1.308615 |
| TG(18:3_18:2_18:3)+NH4 | 892.7389 | 17.20639 | 1.944964 | 0.004246 | 1.161582 |
| TG(18:3_18:2_18:2)+NH4 | 894.7545 | 17.33151 | 1.45588 | 0.001413 | 1.756742 |
| TG(18:1_18:2_18:3)+NH4 | 896.7702 | 17.48404 | 1.265096 | 0.001522 | 2.483677 |
| TG(18:1_18:2_18:2)+NH4 | 898.7858 | 17.61224 | 1.130743 | 0.012901 | 3.161774 |
| LPC(16:1e)-CH3 | 464.3147 | 5.143325 | 0.548588 | 4.76E-09 | 1.640489 |

Supplementary Table S5.

Lipid metabolites altered in plasma of aMCI vs. NC(Analysis of covariance).

| LipidIon | ANCOVA_pvalue |
| --- | --- |
| Cer(d18:1_24:0)+HCOO | 0.065422996 |
| PE(40:9e)-H | 0.04852951 |
| SM(d16:1_16:0)+H | 0.045444066 |
| LPC(20:4)+H | 0.044417877 |
| PI(18:0_20:4)-H | 0.043683613 |
| PE(37:3e)+H | 0.041386079 |
| PE(18:2p_18:2)+H | 0.039075509 |
| LPC(20:3)+Na | 0.030942492 |
| PE(18:1e_18:1)-H | 0.027740332 |
| SM(d18:1_24:2)+H | 0.02258587 |
| PC(34:2)+H | 0.022030524 |
| PC(38:4)+H | 0.021547099 |
| PC(38:5e)+H | 0.017709809 |
| PC(18:0_20:4)+Na | 0.017272533 |
| LPC(18:0)+HCOO | 0.016433041 |
| PE(16:1e_20:4)-H | 0.014976692 |
| PC(32:0e)+H | 0.014554855 |
| SM(d44:5)+H | 0.01415129 |
| Hex1Cer(d18:1_23:0)+HCOO | 0.013977035 |
| PC(18:2_20:4)+HCOO | 0.013550058 |
| SM(d40:1)+HCOO | 0.01194778 |
| PC(34:1e)+H | 0.009918923 |
| TG(18:1_18:2_18:2)+NH4 | 0.009552483 |
| PC(16:0_18:3)+HCOO | 0.009381105 |
| TG(37:6)+Na | 0.008989967 |
| Cer(t17:0_24:0)+HCOO | 0.007646494 |
| PC(16:0_16:0)+Na | 0.007280676 |
| Hex1Cer(d18:1_24:0)+HCOO | 0.006653415 |
| PE(37:2e)+H | 0.005682755 |
| PE(16:0_20:5)-H | 0.004794366 |
| PC(34:2e)+H | 0.003931002 |
| PC(16:1_18:2)+HCOO | 0.003890446 |
| PE(18:2e_20:4)-H | 0.003719099 |
| LPC(20:4)+HCOO | 0.00350261 |
| Cer(d18:1_22:0)+HCOO | 0.003111719 |
| PE(18:0_20:5)-H | 0.003049433 |
| PC(16:0_18:2)+Na | 0.00262578 |
| PE(18:0_18:3)-H | 0.002573769 |
| LPC(18:1)+HCOO | 0.002422212 |
| TG(18:1_18:2_18:3)+NH4 | 0.00145398 |
| TG(18:3_18:2_18:3)+NH4 | 0.001355499 |
| PE(16:1e_18:1)-H | 0.001302311 |
| PC(18:3_18:2)+HCOO | 0.00078987 |
| SPH(d20:0)+H | 0.000772943 |
| Cer(d16:1_24:1)+HCOO | 0.0006681 |
| LPE(20:4)-H | 0.000638577 |
| PE(16:0_18:2)-H | 0.000494832 |
| TG(16:0_10:0_18:1)+NH4 | 0.000286698 |
| TG(18:3_18:2_18:2)+NH4 | 0.000171552 |
| TG(18:0_16:0_16:0)+NH4 | 0.000146701 |
| Cer(m22:1_16:0+O)+HCOO | 0.000143524 |
| PC(18:0_16:0)+HCOO | 0.000114461 |
| TG(16:0_10:1_16:0)+NH4 | 9.64175E-05 |
| PC(16:1e_22:0)+HCOO | 9.02171E-05 |
| PC(16:1_18:2)-CH3 | 5.87915E-05 |
| CL(78:5)-2H | 5.16864E-05 |
| TG(18:0_16:0_18:0)+NH4 | 4.10068E-05 |
| PE(18:0_22:5)-H | 3.75434E-05 |
| PC(18:2_18:2)-CH3 | 3.36458E-05 |
| Cer(d21:0_19:0)+HCOO | 2.34572E-05 |
| TG(16:0_16:0_16:0)+NH4 | 1.39721E-05 |
| PC(15:0_18:2)+H | 1.19134E-05 |
| TG(16:0_10:1_18:1)+NH4 | 5.77065E-06 |
| PC(20:0_11:2)+H | 8.66821E-07 |
| Cer(t17:0_22:0)+HCOO | 7.67458E-07 |
| PE(20:0_20:4)-H | 2.75852E-07 |
| PE(20:1e_20:3)-H | 2.13268E-08 |
| PC(15:0_18:2)+HCOO | 1.07787E-08 |
| LPC(16:1e)-CH3 | 9.86721E-09 |
| Hex1Cer(m40:2)-H | 8.00262E-09 |
| PE(20:1e_20:4)-H | 2.69152E-09 |
| SPH(d22:0)+H | 1.6984E-09 |
| PC(15:0_20:5)+HCOO | 2.98784E-11 |
| SPH(d18:1)+H | 5.03112E-15 |
| SPH(d16:0)+H | 3.47938E-16 |
| SPH(d18:0)+H | 1.13722E-16 |

Adjusted for age, gender, education level

Supplementary Table S6

Identification of potential markers for AD (Adjusted for none)

| LipidIon | Specificity | Sensitivity | AUC(95% CI) |
| --- | --- | --- | --- |
| TG(16:0_10:1_16:0)+NH4 | 0.610 | 0.759 | 0.739(0.645~0.821) |
| TG(16:0_10:1_18:1)+NH4 | 0.458 | 0.852 | 0.685(0.580~0.771) |
| TG(16:0_10:0_18:1)+NH4 | 0.373 | 0.944 | 0.691(0.602~0.785) |
| SPH(d18:0)+H | 0.763 | 0.981 | 0.827(0.727~0.906) |
| SPH(d18:1)+H | 0.814 | 0.926 | 0.847(0.763~0.927) |
| SPH(d22:0)+H | 0.458 | 1.000 | 0.710(0.610~0.807) |
| TG(16:1_12:0_18:1)+NH4 | 0.492 | 0.778 | 0.629(0.531~0.732) |
| SPH(d16:0)+H | 0.949 | 1.000 | 0.985(0.966~1.000) |
| TG(18:0_16:0_18:0)+NH4 | 0.983 | 0.370 | 0.737(0.641~0.817) |
| TG(16:0_16:0_16:0)+NH4 | 0.576 | 0.815 | 0.737(0.628~0.816) |
| TG(18:0_16:0_16:0)+NH4 | 0.576 | 0.741 | 0.719(0.621~0.804) |
| TG(18:0_18:0_18:0)+NH4 | 0.661 | 0.648 | 0.687(0.582~0.788) |
| PC(15:0_18:2)+H | 0.589 | 0.736 | 0.663(0.565~0.757) |
| PC(16:0_18:2)+Na | 0.407 | 0.796 | 0.591(0.478~0.694) |
| TG(18:3_18:2_18:3)+NH4 | 0.407 | 0.852 | 0.621(0.517~0.719) |

Adjusted for none

Supplementary Table S7

Identification of potential markers for AD (Adjusted for age, gender, education level)

| LipidIon | Specificity | Sensitivity | AUC(95% CI) |
| --- | --- | --- | --- |
| TG(16:0_10:1_16:0)+NH4 | 0.628 | 0.801 | 0.761(0.661~0.837) |
| TG(16:0_10:1_18:1)+NH4 | 0.579 | 0.800 | 0.712(0.606~0.802) |
| TG(16:0_10:0_18:1)+NH4 | 0.503 | 0.806 | 0.705(0.600~0.807) |
| SPH(d18:0)+H | 0.589 | 0.955 | 0.755(0.629~0.839) |
| SPH(d18:1)+H | 0.548 | 0.938 | 0.780(0.680~0.853) |
| SPH(d22:0)+H | 0.594 | 0.804 | 0.673(0.573~0.753) |
| TG(16:1_12:0_18:1)+NH4 | 0.447 | 0.812 | 0.645(0.526~0.744) |
| SPH(d16:0)+H | 0.934 | 1.000 | 0.987(0.961~1.000) |
| TG(18:0_16:0_18:0)+NH4 | 0.603 | 0.777 | 0.730(0.624~0.820) |
| TG(16:0_16:0_16:0)+NH4 | 0.691 | 0.705 | 0.738(0.626~0.815) |
| TG(18:0_16:0_16:0)+NH4 | 0.520 | 0.816 | 0.720(0.619~0.808) |
| TG(18:0_18:0_18:0)+NH4 | 0.572 | 0.717 | 0.690(0.592~0.793) |
| PC(15:0_18:2)+H | 0.305 | 0.956 | 0.614(0.518~0.708) |
| PC(16:0_18:2)+Na | 0.391 | 0.839 | 0.606(0.506~0.701) |
| TG(18:3_18:2_18:3)+NH4 | 0.450 | 0.793 | 0.609(0.481~0.725) |

Adjusted for age, gender, education level

Supplementary Table S8

ROC analysis of SPH(d16:0)+H, SPH(d18:1)+H, and SPH(d18:0)+H in the diagnosis of AD

| LipidIon | Specificity | sensitivity | AUC(95% CI) |
| --- | --- | --- | --- |
| joint prediction | 0.949 | 1.000 | 0.990(0.974~1.000) |

Supplementary Table S9

Identification of potential markers for aMCI(Adjusted for none)

| LipidIon | Specificity | sensitivity | AUC(95% CI) |
| --- | --- | --- | --- |
| TG(16:0_10:1_16:0)+NH4 | 0.933 | 0.463 | 0.721(0.608~0.823) |
| TG(16:0_10:1_18:1)+NH4 | 0.333 | 1.000 | 0.694(0.581~0.795) |
| SPH(d18:0)+H | 0.933 | 0.981 | 0.940(0.841~1.000) |
| TG(16:0_10:0_18:1)+NH4 | 0.300 | 0.981 | 0.656(0.535~0.776) |
| SPH(d18:1)+H | 0.933 | 0.926 | 0.934(0.847~0.993) |
| PE(40:9e)-H | 0.222 | 0.980 | 0.524(0.379~0.673) |
| PE(16:1e_20:4)-H | 0.267 | 0.963 | 0.506(0.361~0.640) |
| SPH(d22:0)+H | 0.700 | 0.926 | 0.824(0.722~0.926) |
| SPH(d16:0)+H | 0.900 | 1.000 | 0.936(0.842~1.000) |
| LPC(20:3)+Na | 0.300 | 0.887 | 0.571(0.430~0.692) |
| TG(16:0_16:0_16:0)+NH4 | 0.933 | 0.500 | 0.754(0.644~0.852) |
| SPH(d20:0)+H | 0.567 | 0.870 | 0.641(0.497~0.789) |
| TG(18:3_18:2_18:3)+NH4 | 0.733 | 0.667 | 0.715(0.598~0.827) |
| TG(18:0_16:0_18:0)+NH4 | 0.933 | 0.444 | 0.736(0.623~0.840) |
| TG(37:6)+Na | 0.800 | 0.648 | 0.690(0.572~0.791) |
| PC(16:0_18:2)+Na | 0.767 | 0.537 | 0.674(0.551~0.781) |
| PC(15:0_18:2)+H | 0.793 | 0.679 | 0.757(0.647~0.868) |
| TG(18:0_16:0_16:0)+NH4 | 0.967 | 0.426 | 0.721(0.609~0.821) |
| PE(16:0_20:5)-H | 0.833 | 0.370 | 0.627(0.501~0.745) |
| PC(34:2e)+H | 0.733 | 0.630 | 0.678(0.560~0.802) |
| PC(20:0_11:2)+H | 0.923 | 0.595 | 0.803(0.703~0.894) |
| PE(37:2e)+H | 0.621 | 0.704 | 0.688(0.547~0.809) |
| PC(18:3_18:2)+HCOO | 0.633 | 0.704 | 0.709(0.599~0.810) |

Adjusted for none.

Supplementary Table S10

Identification of potential markers for aMCI (Adjusted for age, gender, education level)

| LipidIon | Specificity | sensitivity | AUC(95% CI) |
| --- | --- | --- | --- |
| TG(16:0_10:1_16:0)+NH4 | 0.451 | 0.943 | 0.756(0.627~0.860) |
| TG(16:0_10:1_18:1)+NH4 | 0.451 | 0.941 | 0.733(0.620~0.834) |
| SPH(d18:0)+H | 0.881 | 0.785 | 0.862(0.689~0.945) |
| TG(16:0_10:0_18:1)+NH4 | 0.451 | 0.869 | 0.695(0.580~0.816) |
| SPH(d18:1)+H | 0.823 | 0.885 | 0.890(0.737~0.962) |
| PE(40:9e)-H | 0.200 | 0.979 | 0.510(0.331~0.653) |
| PE(16:1e_20:4)-H | 0.271 | 0.966 | 0.514(0.378~0.670) |
| SPH(d22:0)+H | 0.805 | 0.658 | 0.742(0.617~0.859) |
| SPH(d16:0)+H | 0.902 | 0.963 | 0.939(0.843~1.000) |
| LPC(20:3)+Na | 0.451 | 0.807 | 0.633(0.483~0.771) |
| TG(16:0_16:0_16:0)+NH4 | 0.751 | 0.704 | 0.791(0.691~0.884) |
| SPH(d20:0)+H | 0.458 | 0.823 | 0.570(0.419~0.703) |
| TG(18:3_18:2_18:3)+NH4 | 0.721 | 0.754 | 0.747(0.630~0.846) |
| TG(18:0_16:0_18:0)+NH4 | 0.767 | 0.654 | 0.775(0.674~0.870) |
| TG(37:6)+Na | 0.749 | 0.566 | 0.671(0.553~0.791) |
| PC(16:0_18:2)+Na | 0.819 | 0.498 | 0.659(0.552~0.792) |
| PC(15:0_18:2)+H | 0.886 | 0.462 | 0.693(0.571~0.799) |
| TG(18:0_16:0_16:0)+NH4 | 0.942 | 0.445 | 0.752(0.645~0.847) |
| PE(16:0_20:5)-H | 0.431 | 0.833 | 0.649(0.510~0.767) |
| PC(34:2e)+H | 0.659 | 0.638 | 0.633(0.514~0.763) |
| PC(20:0_11:2)+H | 0.846 | 0.487 | 0.702(0.606~0.828) |
| PE(37:2e)+H | 0.717 | 0.584 | 0.625(0.521~0.755) |
| PC(18:3_18:2)+HCOO | 0.779 | 0.577 | 0.716(0.592~0.823) |

Adjusted for age, gender, education level

Supplementary Table S11

ROC analysis of SPH(d16:0)+H, SPH(d18:1)+H, and SPH(d18:0)+H in the diagnosis of aMCI

| LipidIon | Specificity | sensitivity | AUC(95% CI) |
| --- | --- | --- | --- |
| joint prediction | 0.900 | 0.981 | 0.934(0.860~0.999) |

Supplementary Table S12

Correlation between lipid metabolites and oxidative stress

|  | MDA | SOD | GSH-PX |
| --- | --- | --- | --- |
| SPH(d16:0)+H | 0.180* | -0.243** | -0.096 |
| SPH(d18:0)+H | 0.227** | -0.330** | -0.109 |
| PC(15:0_20:5)+HCOO | -0.125 | 0.318** | 0.123 |
| PC(15:0_18:2)+HCOO | -0.149 | 0.269** | 0.045 |
| PC(16:0_18:2)+Na | 0.152 | -0.145 | -0.129 |
| SPH(d22:0)+H | 0.131 | -0.314** | -0.073 |
| PE(20:1e_20:4)-H | -0.168* | 0.318** | 0.299** |
| Cer(d21:0_19:0)+HCOO | -0.102 | 0.223** | -0.13 |
| TG(18:0_16:0_16:0)+NH4 | 0.033 | -0.362** | -0.078 |
| LPC(16:1e)-CH3 | -0.153 | 0.225** | -0.015 |
| PE(20:0_20:4)-H | -0.149 | 0.13 | -0.078 |
| PC(15:0_18:2)+H | -0.017 | -0.340** | -0.228** |
| TG(16:0_16:0_16:0)+NH4 | -0.011 | -0.292** | -0.068 |
| TG(18:0_16:0_18:0)+NH4 | 0.038 | -0.356** | -0.105 |
| SPH(d18:1)+H | 0.132 | -0.342** | -0.075 |
| PE(20:1e_20:3)-H | -0.043 | 0.237** | 0.175* |
| TG(18:3_18:2_18:3)+NH4 | 0.132 | -0.202* | -0.183* |
| TG(16:0_10:1_18:1)+NH4 | -0.032 | -0.148 | -0.067 |
| TG(16:0_10:0_18:1)+NH4 | -0.043 | -0.151 | -0.056 |
| Hex1Cer(m40:2)-H | -0.128 | 0.201* | -0.036 |
| TG(16:0_10:1_16:0)+NH4 | -0.017 | -0.156 | -0.087 |

Partial correlation analysis (adjusted for education level, age, gender .* p < 0.05; ** p < 0.01.

Supplementary Table S13

Correlation between lipid metabolites and oxidative stress

|  | MDA | SOD | GSH-PX |
| --- | --- | --- | --- |
| MMSE | -0.192* | 0.284** | -0.192* |
| MoCA | -0.210* | 0.291** | -0.193* |
| immediate recall | -0.348** | 0.308** | -0.123 |
| forward digit span | -0.143 | 0.049 | -0.143 |
| backward digit span | -0.192* | 0.102 | -0.131 |
| BNT | -0.098 | 0.324** | -0.163 |
| delayed recall | -0.332** | 0.338** | -0.038 |
| long delayed recognition | -0.332** | 0.308** | -0.08 |

Partial correlation analysis (adjusted for education level, age, gender .* p < 0.05; ** p < 0.01.
